# Structure–Function Coupling in Pyridyl Triazole Copolymers for Neuromorphic Synaptic Transistors

**DOI:** 10.1021/acsaelm.5c02633

**Published:** 2026-02-12

**Authors:** Arash Ghobadi, Salahuddin Attar, Abhijeet Abhi, Thomas B. Kallaos, Dilan M. Gamachchi, Indeewari M. Karunarathne, Andrew C. Meng, Joseph C. Mathai, Shubhra Gangopadhyay, Steven P. Kelley, Mohammed Al-Hashimi, Suchismita Guha

**Affiliations:** 1 Department of Physics and Astronomy, 14716 University of Missouri, Columbia, Missouri 65211, United States; 2 College of Science and Engineering, 370593 Hamad Bin Khalifa University, Doha 34110, Qatar; 3 MU Materials Science and Engineering Institute, 14716 University of Missouri, Columbia, Missouri 65211, United States; 4 Department of Electrical Engineering and Computer Science, 14716 University of Missouri, Columbia, Missouri 65211, United States; 5 Department of Chemistry, 14716 University of Missouri, Columbia, Missouri 65211, United States

**Keywords:** donor−acceptor polymers, organic transistor, neuromorphic device, neural network, trap density of states

## Abstract

Organic ferroelectric transistors are excellent candidates as low-cost alternatives for synaptic devices. Specifically, interfaces with donor–acceptor semiconducting polymers and copolymers of poly­(vinylidene fluoride) (PVDF) are attractive for mimicking synaptic responses. By tailoring the linking unit between the pyridyl triazole (PyTr) acceptors and thiophene donors, three copolymers are synthesized incorporating selenium-substituted thiophene, benzothiadiazole, and fluorine-substituted thiophene linkers. Using the hexafluoropropylene copolymer of PVDF (PVDF-HFP) as the dielectric layer, the three PyTr semiconductors show p-type transport in transistor architectures with carrier mobilities between 0.1 and 0.2 cm^2^ V^–1^ s^–1^. The synaptic plasticity is investigated by applying long-term pulsed voltages at the gate electrode to emulate potentiation and depression processes. To assess their neuromorphic functionality, the synaptic responses of the devices are tested for image recognition in a multilayer perceptron neural network. The copolymer with the benzothiadiazole linker achieved recognition accuracy close to 80%, whereas the one with a fluorine-substituted thiophene linker shows no synaptic behavior, highlighting the critical role of the semiconductor–dielectric interface. A detailed study of the interface trap density and morphology is performed to identify how interfacial properties directly influence synaptic device performance.

## Graphical abstract

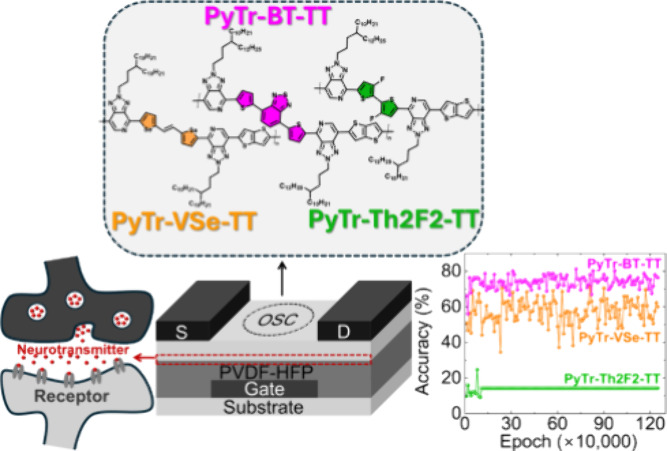

## Introduction

Plastic electronics utilizing organic semiconductors have revolutionized technology since the 1990s. Wearable, flexible, lightweight displays and solar cells based on organic semiconductors have now become a reality.^1–3^ Conjugated polymers and organic molecules continue to attract widespread attention because of their promise for low-cost, large-area optoelectronic and photonic device applications. More recently, organic transistors based on π-conjugated systems have evolved as excellent candidates for emulating synaptic characteristics, thus opening new avenues in brain-like computing with the potential of achieving exceptional efficiency and low energy consumption.^4–8^

Approximately 10^15^ synapses regulate communication between neurons and form the basis of learning and memory through activity-dependent modulation of their connection strength, a phenomenon known as synaptic plasticity. Drawing inspiration from these mechanisms, neuromorphic computing has emerged,^9,10^ mimicking neural network architectures that integrate memory and processing to enable massively parallel and energy-efficient computation. This paradigm is driving the development of bioinspired electronics for next-generation intelligent systems, motivated by the increasing demand for efficient and human-like machines.^11–14^ Building on this foundation, artificial synaptic devices have been engineered to reproduce key plasticity mechanisms, including short-term and long-term plasticity, adaptation, spike-timing-dependent plasticity (STDP), and excitatory and inhibitory postsynaptic currents (EPSC/IPSC). These devices successfully emulate synaptic responses such as short-term potentiation and depression (STP/STD), long-term potentiation and depression (LTP/LTD), and paired-pulse facilitation (PPF), allowing the realization toward energy-efficient hardware platforms capable of supporting advanced applications in perception, memory, and pattern recognition.

Synapses serve as the fundamental units of neural communication, connecting presynaptic and postsynaptic neurons, as shown in Figure 1a. Chemical synapses release neurotransmitters, which bind to receptors on the postsynaptic neuron, modulating the postsynaptic current. The strength of a synapse in biological systems can be gradually enhanced or diminished depending on the timing of spikes between the presynaptic and postsynaptic neurons, as well as the neuron’s biological potential and the type and quantity of neurotransmitters involved. Organic thin-film transistors have emerged as promising alternatives for artificial synaptic devices. Compared with two-terminal memristor-type devices, they offer a clear advantage in terms of low power consumption.^14–16^ Similar to a presynaptic neuron, the gate terminal modulates the channel conductance. The postsynaptic terminal, consisting of the source and drain electrodes within the active layer, is modulated in a way analogous to synaptic weight, influencing charge storage and transmission (see Figure 1b). The resulting changes in the source-drain current represent postsynaptic response, reproducing the plasticity and functional behavior of biological synapses. The conducting channel of the transistor acts as the synaptic cleft, the narrow space where the neurotransmitters interact with receptors.

**Figure 1:**
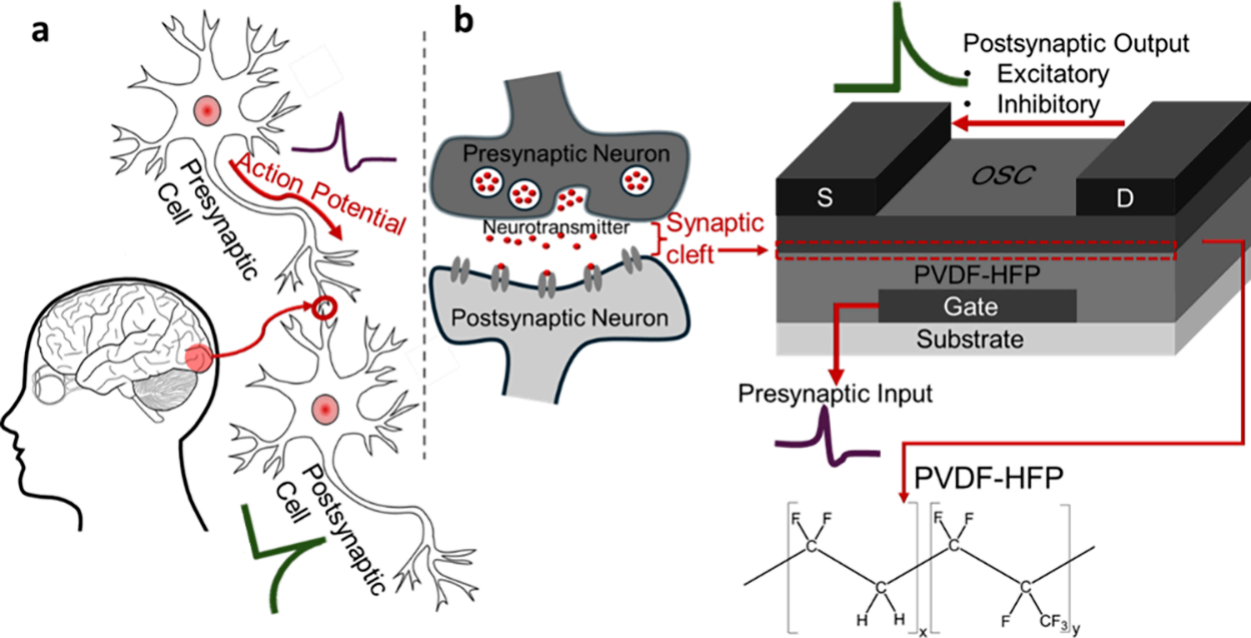
(a) Schematic of a biological synapse. (b) Architecture of a bottom-gate, top-contact FET and its analogous functionality to a pre- and postsynaptic neuron. The organic semiconductor–ferroelectric interface emulates the synaptic cleft where the electronic conductance is modulated. Presynaptic input is applied to the gate electrode, and the postsynaptic output is determined from the source-drain current. PVDF-HFP is used as the dielectric layer.

To emulate synaptic characteristics, the multiconductance states can be achieved through several mechanisms, for instance, using a ferroelectric dielectric in a field-effect transistor (FET) architecture, ion-driven (de)­doping as in organic electrochemical transistors (OECT), or through combined ionic–electronic transport as seen in electrochemical random access memory (EC-RAM) devices.^14,17^ Among these, OECTs and ion-gated transistors are particularly attractive as low-voltage synaptic devices; here, the active channel directly interfaces with an electrolyte and conductance modulation arises from ionic (de)­doping processes.^13,18,19^ Despite notable advances, important questions remain regarding how electrolyte composition, ion mobility, and transient response collectively influence device performance and what design strategies yield the most efficient architectures.

Organic ferroelectric FETs with their simple architecture where both the semiconducting and ferroelectric layers may be solution-processed (using orthogonal solvents) offer a versatile platform for realizing electrical and photonic synapses.^20,21^ Side-chain modulation of conjugated polymers is also seen to play a role in the synaptic response from transistors.^22^ Ferroelectric polymers derived from polyvinylidene fluoride (PVDF) are widely used as dielectric layers due to their ability to switch between multiple polarization states under an external electric field, enabling precise modulation of carrier concentration in the semiconductor channel.^23–26^ Copolymers such as PVDF with trifluoroethylene (PVDF-TrFE) and PVDF hexafluoropropylene (PVDF-HFP) are among the most commonly used ferroelectric materials in synaptic transistors. While PVDF-TrFE exhibits strong remanent and saturation polarization characteristic of classical ferroelectrics, PVDF-HFP behaves as a relaxor ferroelectric with reduced long-range dipole ordering.^27^ Despite the weak polarization effects in PVDF-HFP, organic FETs incorporating this dielectric exhibit a pronounced response.^20,28^ Using a donor–acceptor copolymer based on diketopyrrolopyrrole (DPP-DTT) and comparing devices fabricated with PVDF-TrFE and PVDF-HFP, we have recently shown that the underlying mechanism of synaptic plasticity differs in the two copolymers.^29^ While the synaptic response in PVDF-TrFE-based organic FETs is governed by ferroelectric domain switching, the synaptic response in PVDF-HFP-based devices is mainly due to ion motion within the dielectric, as ferroelectric polarization can be influenced by both electronic and ionic conduction. The migration of ions at the semiconductor–dielectric interface dynamically modulates the channel conductance, enabling the emulation of key synaptic functions. Given that both the semiconductor and dielectric layers are solution-processed without any interface modification, an important question arises regarding the extent to which the organic semiconductor itself plays a role in the synaptic weight update, especially when ions are involved as in PVDF-HFP. To address this, in this work, we investigate three donor–acceptor copolymers based on pyridyl triazole, each with distinct backbone architectures.

Donor–acceptor copolymers have played a central role in advancing organic photodetectors, particularly those operating in the near-infrared (IR) region.^30–32^ Their optical and electronic properties can be effectively tuned through the incorporation of electron-deficient and electron-rich units. Acceptor groups such as diketopyrrolopyrrole (DPP)^33–35^ and isoindigo (IID),^36–38^ which yield narrow optical band gaps extending from the red to the IR region, have demonstrated excellent charge transport characteristics in organic transistors. More recently, pyridyl triazole (PyTr)-based donor–acceptor copolymers have gained significant interest, as their backbone coplanarity can be tailored by the linking units between the donor and the acceptor groups. In particular, PyTr acceptor units with thiophene-based donor groups show full-backbone coplanarity due to inter- and intramolecular noncovalent interaction,^39^ leading to enhanced charge transport and luminescence, as well as suitability for stretchable electronic applications.^40,41^

This study introduces three unique donor–acceptor copolymers incorporating PyTr-based acceptor units featuring (a) an (*E*)-1,2-di­(selenophen-2-yl)­ethene-vinylidene (VSe)-bridged PyTr acceptor paired with a coplanar thieno­[3,2-*b*]­thiophene (TT) donor (PyTr-VSe-TT), (b) 4,7-di­(thiophen-2-yl)­benzo­[*c*]­[1,2,5]­thiadiazole (BT)-bridged PyTr acceptor with a coplanar TT donor (PyTr-BT-TT), and (c) 3,3′-difluoro-2,2′-bithiophene (BiThF2)-bridged PyTr acceptor with a coplanar TT donor (PyTr-Th2F2-TT). The bridging unit provides the tuning of optical properties due to their varying donor–acceptor property. The synthetic routes to the bridged bis-PyTr monomers (**M1**, **M2**, and **M3**) and their polymers are shown in Scheme 1 and Scheme 2, respectively.

**Scheme 1:**
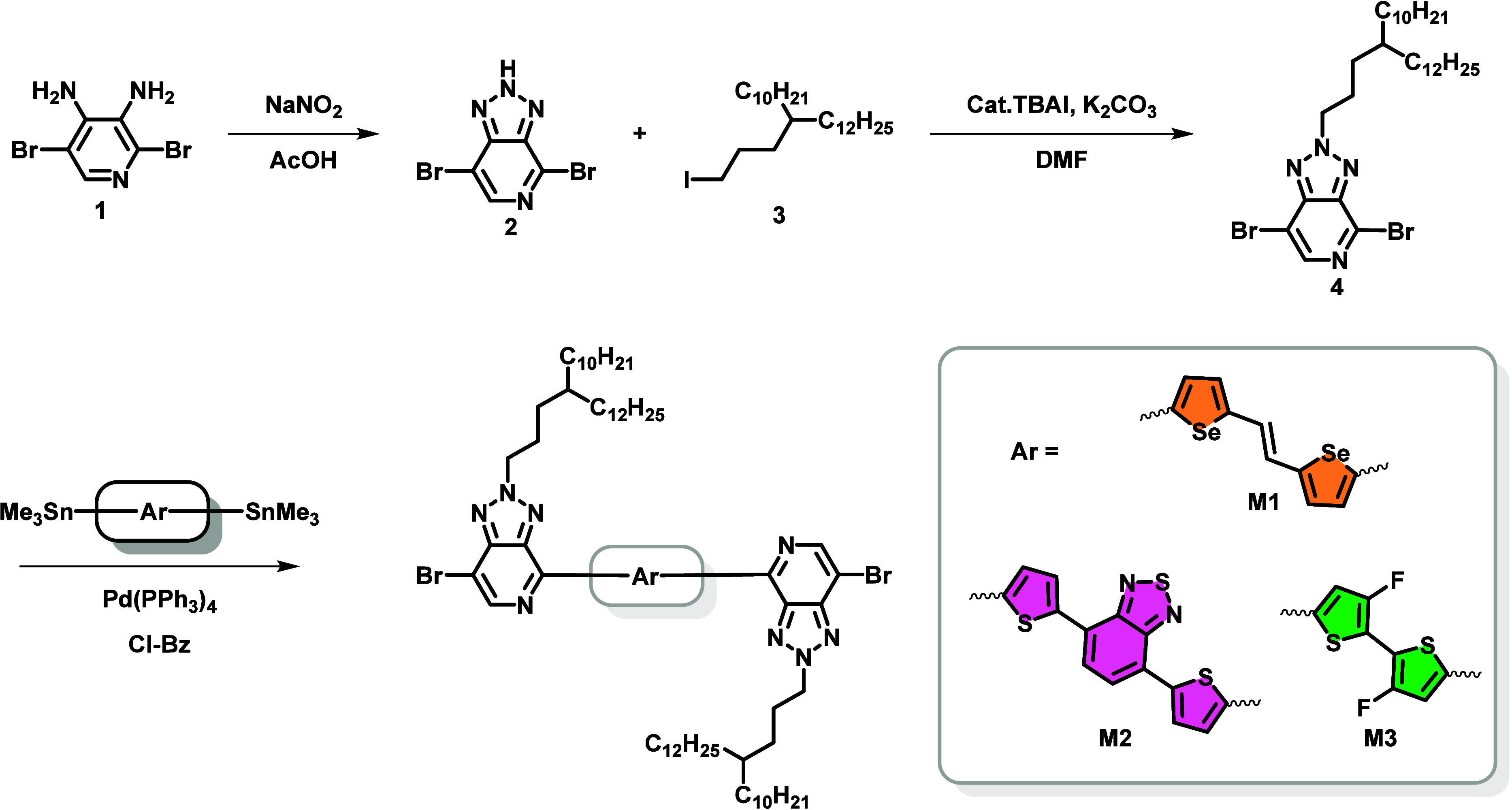
Synthesis of Monomers

**Scheme 2:**
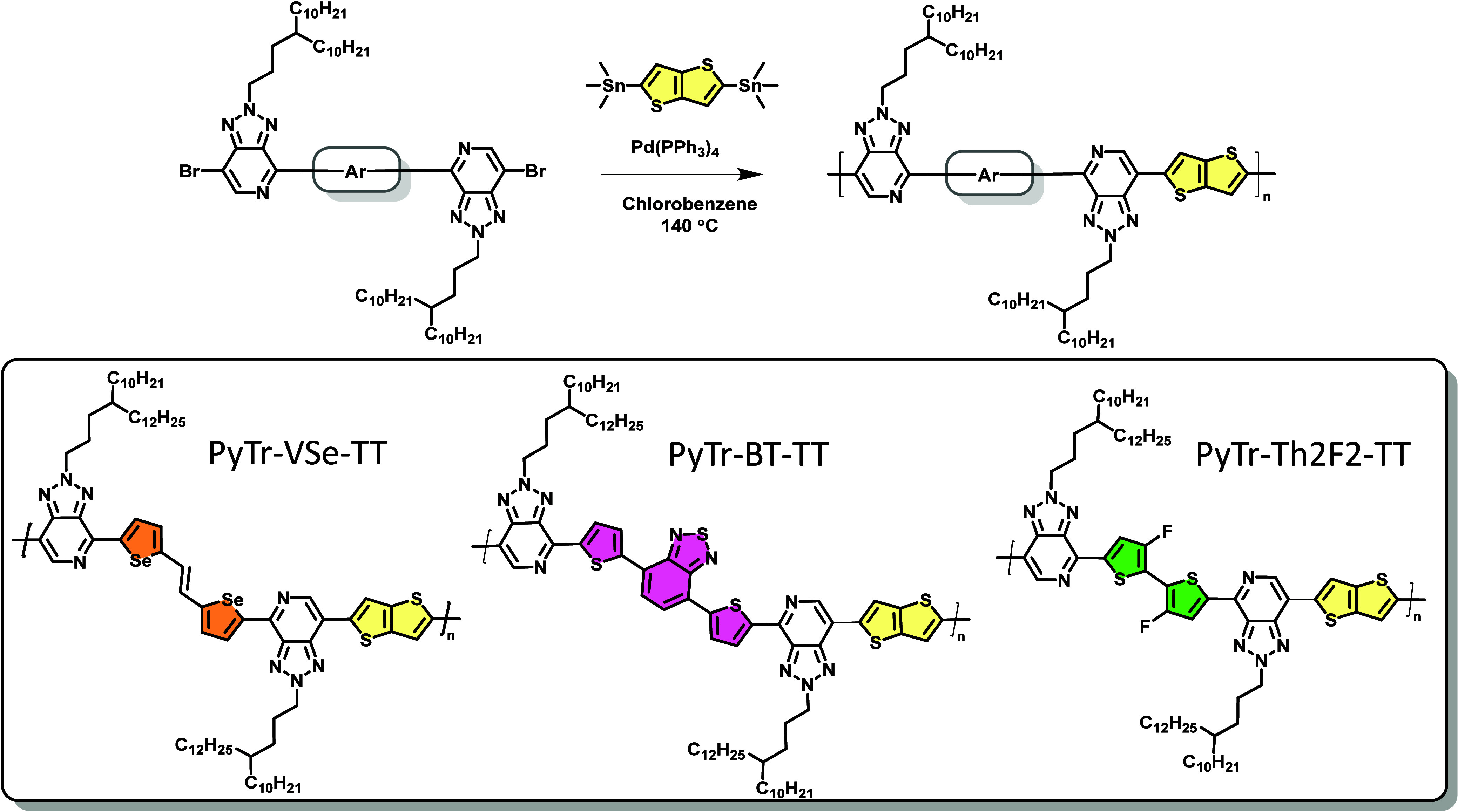
Synthesis of PyTr Copolymers

Each copolymer was employed as the active semiconducting layer in bottom-gate, top-contact organic FETs with PVDF-HFP serving as the gate dielectric. Synaptic plasticity was investigated by applying long-term pulsed voltages of around 200 pulses at the gate electrode to emulate potentiation and depression processes, enabling the evaluation of the synaptic weight in the LTP and LTD cycles. The resulting changes in channel conductance displayed a nonlinear dependence on the sequence of potentiation and depression pulses, confirming their ability to reproduce artificial synaptic behavior. Due to the remnant polarization of PVDF-based dielectrics, STP/STD behavior is usually not seen in organic FETs when the interface comprises just the organic semiconducting and PVDF layers. The all-organic channel and dielectric interface, together with slow polarization dynamics, produce conductance states that persist far longer than the time scales typically associated with short-term plasticity (STP/STD) and PPF. Hence, we focus only on the LTP/LTD response. To assess their neuromorphic functionality, the devices were further tested for image recognition tasks using a multilayer perceptron neural network trained on 20 × 20 pixel handwritten digits from the MNIST (Modified National Institute of Standards and Technology) database through the NeuroSim framework.^42^ Among the three copolymers, devices based on PyTr-BT-TT achieved recognition accuracy close to 80%. While all devices exhibited broadly similar electrical characteristics, only PyTr-VSe-TT and PyTr-BT-TT showed synaptic performance, highlighting the critical role of the semiconductor–dielectric interface. Fluorine substitution in PyTr-Th2F2-TT hinders ionic motion and shows no synaptic behavior. A detailed study of trap density and interfacial morphology was performed to clarify the origin of these differences and demonstrate how interfacial properties directly influence synaptic device performance.

## Results and Discussion

Detailed synthesis procedures for the three copolymers are provided in the Supporting Information. Here, we provide a brief description. The synthetic sequence for preparation of monomers is reported in Scheme 1. Stille polymerization was successfully performed by reacting the stannylated monomers with Ar-bridged PyTr dibromides in the presence of 2 mol % tetrakis­(triphenylphosphine)­palladium(0) [Pd­(PPh_3_)_4_] as a catalyst in refluxing chlorobenzene for 8 h. The resulting dark solid polymers were obtained after precipitation in acidic methanol (Scheme 2) and were subjected to sequential Soxhlet extraction using methanol, acetone, hexane, chloroform, and chlorobenzene for 8 h to remove low-molecular-weight oligomers and residual catalyst. The pure chlorobenzene fraction obtained was then used for all the characterization and device fabrication. The molecular weights and polydispersity (PDI) values are reported in Table S1.

The relatively high molecular weight of PyTr-Th2F2-TT can be attributed to its more planar and rigid conjugated backbone, arising from noncovalent S–F interactions and a lower number of rotatable bonds (three) along the conjugated backbone. This structural rigidity facilitates efficient π–π stacking and promotes effective chain propagation during polymerization. In contrast, PyTr-VSe-TT and PyTr-BT-TT incorporating VSe and thiophene BT linkers contribute a higher number of rotatable bonds (four) in a single unit, leading to increased conformational flexibility and a greater tendency toward nonplanar conformations. Such structural features can hinder effective chain growth during polymerization, resulting in comparatively lower molecular weights.

### Optical and Electrochemical Properties

The optoelectronic properties of conjugated polymers are dictated by their molecular architecture and supramolecular organization. Upon transitioning from solution to the solid-state film, notable variations arise in their optical absorption profiles, primarily due to the development of interchain interactions and collective excitonic coupling. As shown in Figure 2a–c, the UV–vis absorption spectra of the three copolymers in the solution phase and in films exhibit strong absorption in the visible to near-infrared region (450–800 nm). The dominant absorption bands centered around 650–710 nm are due to the intramolecular charge transfer transition between the donor and acceptor moieties. Notably, PyTr-Th2F2-TT with the highest molecular weight (*M*_n_ = 224 kDa) shows slightly blueshifted absorption compared to PyTr-VSe-TT and PyTr-BT-TT, suggesting that the higher molecular weight and PDI (*Đ* = 2.7) may introduce chain entanglement or structural disorder, limiting effective backbone planarity. In contrast, the moderate molecular weight and narrower PDI in PyTr-VSe-TT and PyTr-BT-TT favor more ordered packing and efficient π–π stacking. The films display well-defined vibronic structures, signifying enhanced interchain π–π stacking, increased backbone planarity, and possible J-aggregate-type ordering, which collectively contribute to improved electronic coupling in the solid state. While PyTr-BT-TT displays a slight redshift in its film compared to its solution spectrum, the overall absorption profile remains similar, suggesting limited aggregation in the solid state relative to the PyTr-VSe-TT and PyTr-Th2F2-TT films.

**Figure 2:**
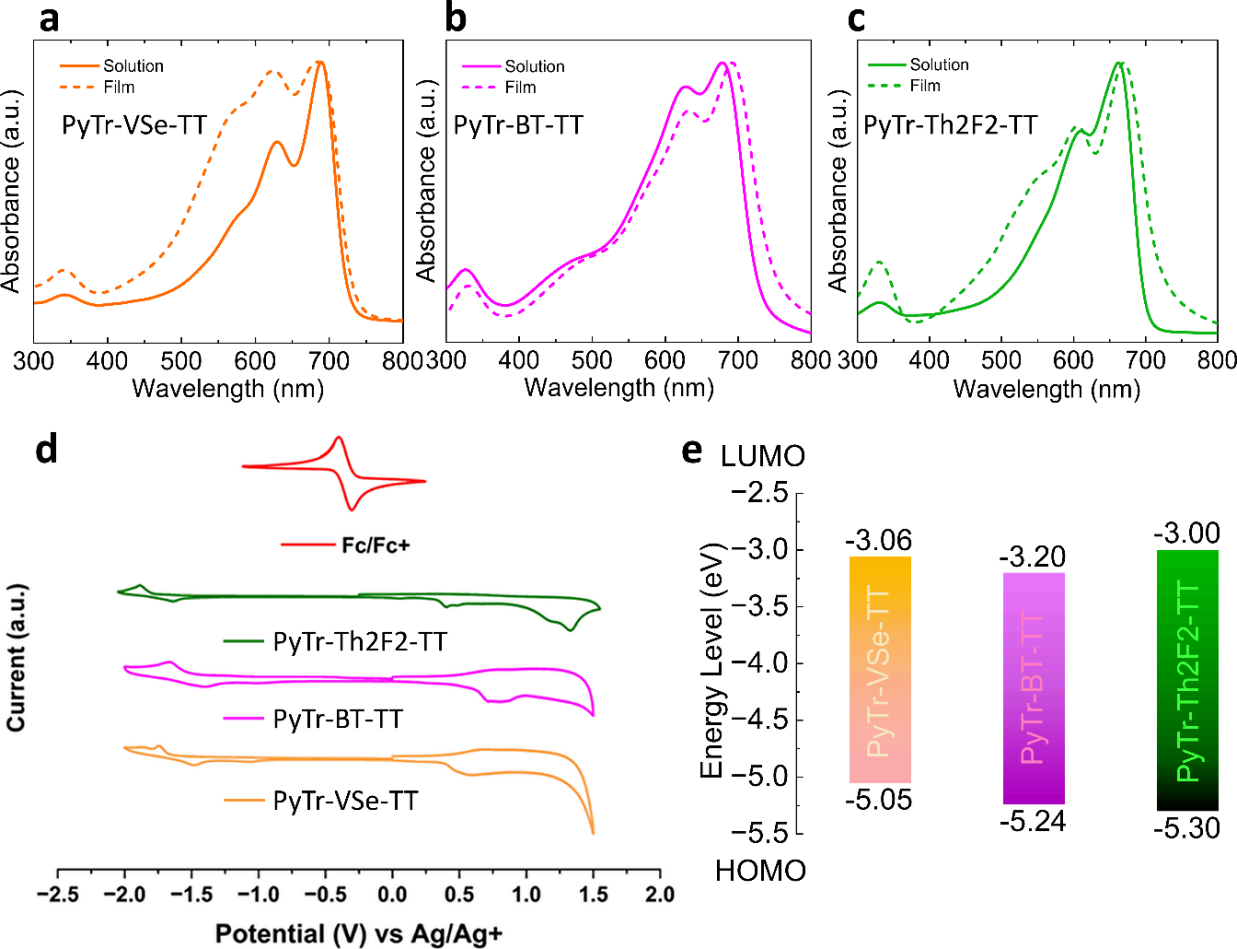
(a–c) Normalized UV–vis absorption spectra of PyTr-VSe-TT, PyTr-BT-TT, and PyTr-Th2F2-TT from chlorobenzene solution (solid line) and thin films (dotted line). (d) Cyclic voltammograms used to estimate the frontier molecular orbital energy levels in (e).

The absorption maxima (λ_max_) of the solution and film of all three copolymers are tabulated in Table S1. The optical band gaps (*E*_g_^opt^) calculated from the absorption onsets range narrowly from 1.62 eV (PyTr-BT-TT) to 1.69 eV (PyTr-Th2F2-TT). Electrochemically, the polymers exhibit lowest unoccupied molecular orbital (LUMO) energies from −3.0 eV (PyTr-BT-TT) to −3.2 eV (PyTr-Th2F2-TT) and highest occupied molecular orbital (HOMO) energies ranging from −5.30 eV (PyTr-Th2F2-TT) to −5.05 eV (PyTr-VSe-TT) (Figure 2e,f). The electrochemical band gaps (*E*_g_^opt^) generally align with the optical data (Table S1). These values further indicate that both absorption and redox properties can be tuned by modifying the acceptor bridged monomers (Scheme 1), critical for optimizing the performance in electronic devices.

### Thermal Properties

All three PyTr-based copolymers demonstrate high thermal stability with thermal decomposition temperature *T*_onset_ values in the range of 414–436 °C and *T*_max_ values between 464 and 489 °C, confirming their robustness under elevated temperatures (Figure S16). PyTr-VSe-TT shows an onset decomposition at 414 °C with a *T*_max_ of 464 °C, undergoing a major mass loss of 61.43% and leaving 38–40% char yield at 600 °C. PyTr-BT-TT, incorporating the electron-deficient BT bridging unit, exhibits slightly enhanced stability with *T*_onset_ = 420 °C and a significantly higher *T* ^max^ of 478 °C. Notably, PyTr-BT-TT leaves a substantially higher char yield (49–50%), consistent with its benzothiadiazole-enriched aromatic conjugation, which favors carbonaceous residue formation. PyTr-Th2F2-TT displays the highest thermal stability among the three materials, with *T*_onset_ = 436 °C and *T*_max_ = 489 °C, reflecting the strong stabilizing effect of the difluoro-bithiophene bridge. Fluorination is known to enhance backbone planarity through noncovalent F···S and F···H interactions, which increases packing rigidity and suppresses early-stage thermal decomposition. The mass losses in PyTr-Th2F2-TT (59.75%) and its char residue (39–41%) are comparable to PyTr-VSe-TT, consistent with polymers dominated by fused aromatic heterocycles. The overall thermal stability trend follows PyTr-Th2F2-TT > PyTr-BT-TT > PyTr-VSe-TT in both *T*_onset_ and *T*_max_. This trend highlights the relative contributions of the electronic structure and backbone rigidity. PyTr-Th2F2-TT benefits from fluorine-induced conformational locking and stronger intermolecular interactions; PyTr-BT-TT benefits from the electron-withdrawing and rigid BT core; and PyTr-VSe-TT, although thermally stable, contains heavier selenium atoms with intrinsically weaker C–Se bonds, leading to slightly earlier decomposition. All three materials exhibit high char yields (38–50%), characteristic of conjugated aromatic polymers and beneficial for applications requiring thermal endurance, such as high-temperature processing of thin films or device fabrication steps involving thermal annealing. These results confirm that backbone engineering via the bridging unit provides an effective strategy to tune both thermal stability and structural integrity in donor–acceptor copolymers.

The differential scanning calorimetry curves for the three copolymers are shown in Supporting Information (Figure S17). They all exhibit initial endothermic events that are visible in the low temperature range, followed by stable heat flow during subsequent heating and cooling, indicating thermal stability and the absence of major phase transitions. There are prominent endothermic peaks at the beginning of the heating ramps (around 30–50 °C) mostly for PyTr-VSe-TT and PyTr-BT-TT, likely corresponding to the removal of moisture or dehydration, or possibly a glass transition or melting event. After the initial peaks, the curves flatten and then decline, reflecting the heat capacity of the sample as it is steadily heated. The cooling curves mirror the heating curves, with gradual reductions in heat flow, but do not show dramatic crystallization peaks, possibly indicating amorphous behavior or high thermal stability. The lack of sharp exothermic or endothermic events at higher temperatures suggests that the three copolymers do not undergo significant structural changes or decomposition within the temperature range of 30–300 °C.

### Transistor Performance and Synaptic Response

Prior to evaluating the synaptic performance of the transistors, their basic electrical characteristics were examined. All measurements were carried out under ambient conditions. Figure 3a–f shows typical FET output and transfer characteristics for the three copolymers with PVDF-HFP as the dielectric layer. The current–voltage characteristics from at least 10 different FETs of each copolymer were measured. The output and transfer characteristics of another set of PyTr-VSe-TT and PyTr-Th2F2-TT FETs with *W*/*L* = 20 are shown in Figure S18. All devices operate below 8 V and exhibit comparable saturation carrier mobilities in the range of 0.1–0.2 cm^2^/(V s). The average values of the FET performance characteristics based on the three copolymers are shown in Table 1.

**Figure 3:**
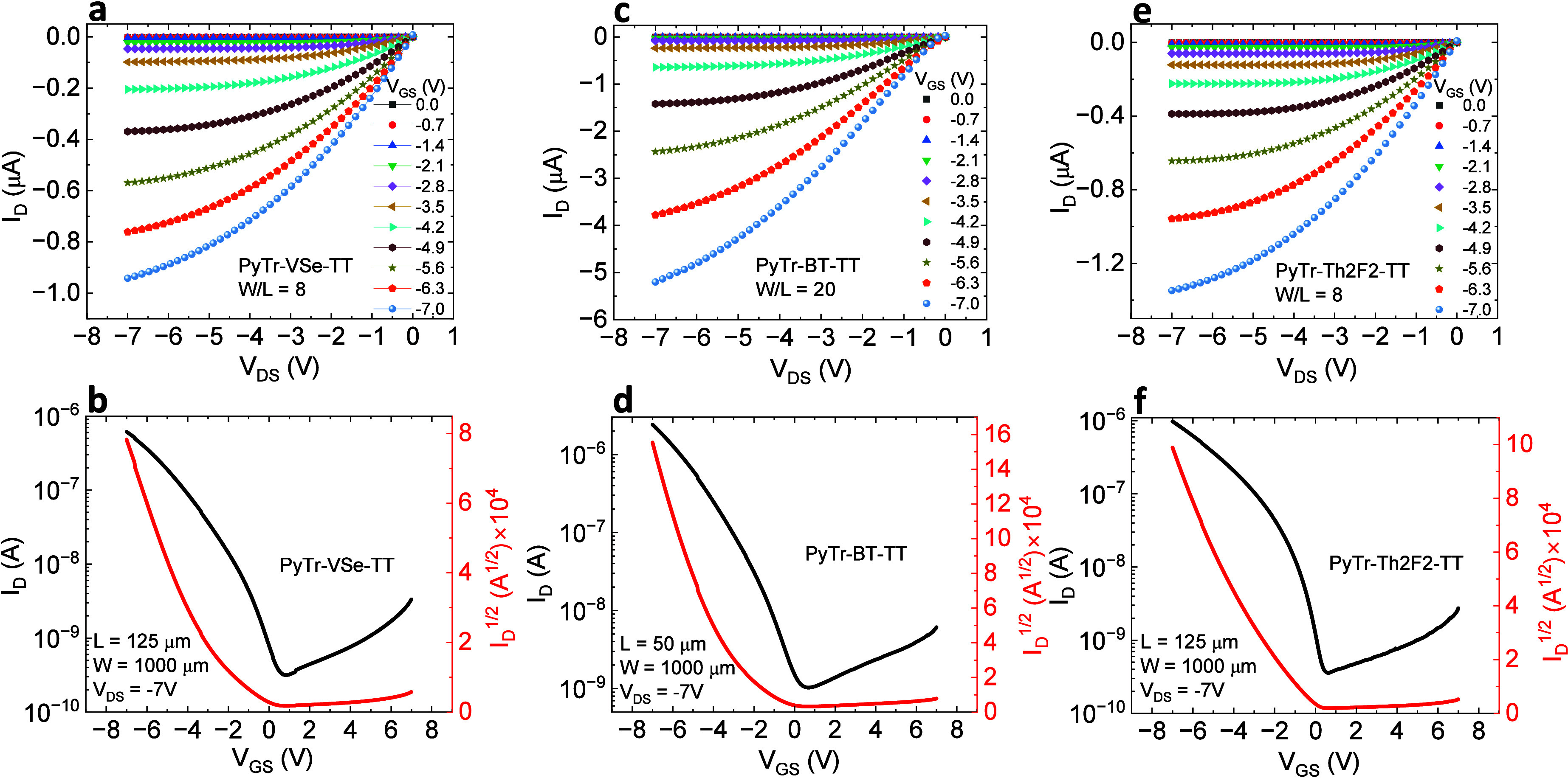
Output and transfer characteristics from PyTr-VSe-TT FET (a, b), PyTr-BT-TT FET (c, d), and PyTr-Th2F2-TT FET (e, f).

**Table 1:** Average Values of Carrier Mobility, Threshold Voltage, and Subthreshold Swing from FETs Based on the Three Copolymers^a^

semiconductor	mobility (cm^2^/(V s))	*V*_th_ (V)	SS (V/decade)
PyTr-VSe-TT	0.12 ± 0.04 (0.18)	–2.75 ± 0.16 (−2.49)	1.81 ± 0.32 (1.28)
PyTr-BT-TT	0.12 ± 0.04 (0.19)	–3.36 ± 0.22 (−2.98)	1.46 ± 0.14 (1.25)
PyTr-Th2F2-TT	0.09 ± 0.04 (0.14)	–2.17 ± 0.27 (−1.69)	0.82 ± 0.75 (0.076)

a The values in parentheses are the best values.

To emulate LTP/LTD behavior, a train of pulses was applied to the gate terminal, and the source-drain voltage was held at −7 V. All three copolymers showed only p-type transport; hence, the potentiating pulse was negative, and the depressing pulse was positive. The timing of the pulses was 1 s on and 2 s off. The pulses during LTP and LTD result in EPSC and IPSC, as shown in Figure 4a,b for a PyTr-BT-TT FET. The insets represent how the conducting channel of a transistor is analogous to the postsynaptic cell connected to the soma of a neuron. As the current was measured using a lock-in amplifier, the displayed current values are higher than the actual values by the gain factor, which was set to 100 for most measurements. The magnitude of the depressing voltage pulse was kept lower than in potentiation pulses to ensure that the maximum conductance during LTD matched the saturation value observed during LTP. Although the signal is noisy, the EPSC and IPSC can be discerned over the general noise level, as seen in the zoomed-in regions in Figure S20. Device-to-device comparison and details of consecutive measurements from the same device are provided in Table S2.

**Figure 4:**
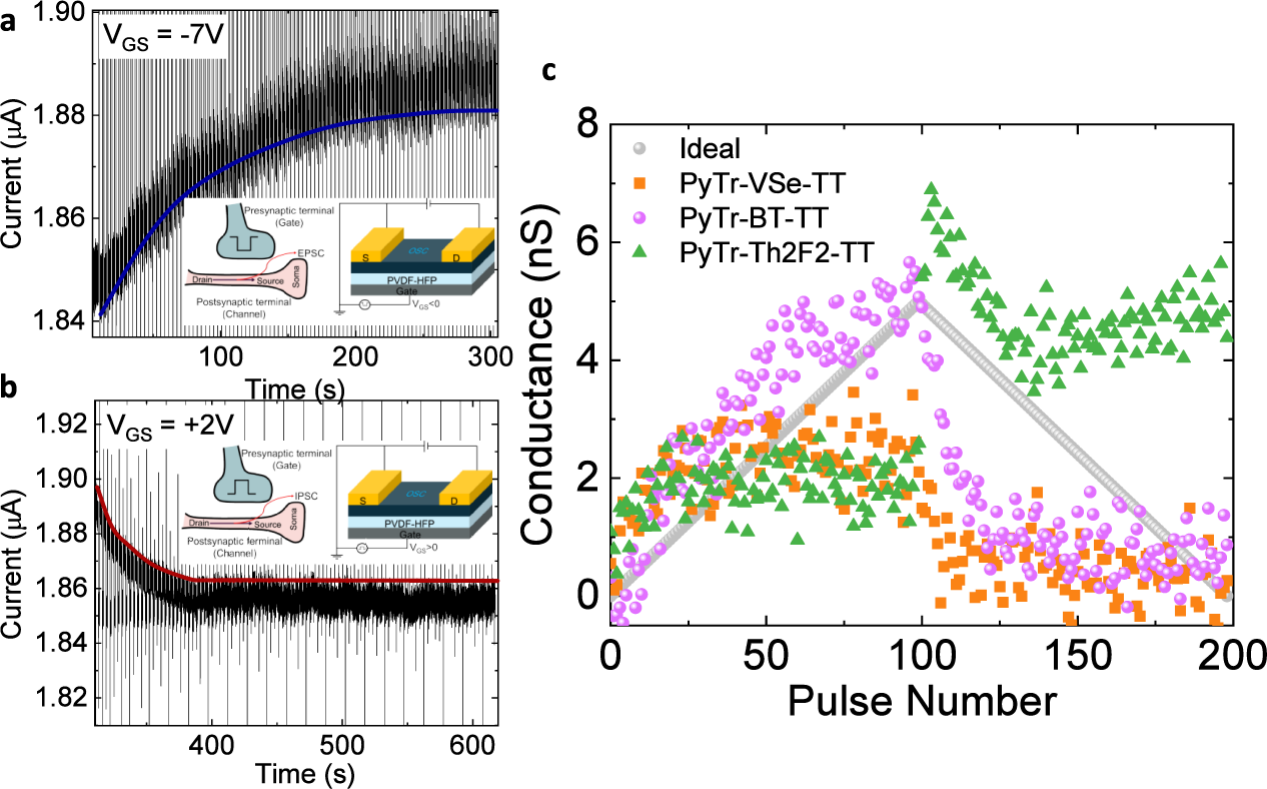
Postsynaptic output current in PyTr-BTT-TT/PVDF-HFP FET during (a) potentiating pulses of −7 V and (b) depressing pulses of 2 V. The insets show the biasing of the FETs with an analogy to a post- and presynaptic neuron. (c) Conductance states from three representative FETs with PyTr-VSe-TT, PyTr-BT-TT, and PyTr-Th2F2-TT as the active semiconducting layer. The gray symbol depicts the synaptic weight uptake for an ideal ferroelectric FET.

The conductance as a function of the pulse number for three individual FETs using PyTr-VSe-TT, PyTr-BT-TT, and PyTr-Th2F2-TT as the semiconducting layer and PVDF-HFP as the dielectric is shown in Figure 4 c. Although PyTr-Th2F2-TT performs well as a p-type FET with PVDF-HFP and exhibits partial response in LTP, no LTD behavior is observed. The PyTr-BT-TT FET demonstrates the strongest synaptic response among the three. For reference, the gray symbols in Figure 4c represent the conductance behavior of an ideal ferroelectric FET, in which the weight increase during LTP and the decrease during LTD follow a linear trend. In contrast, real devices typically deviate from this ideal linearity, showing a steep increase in conductance during the initial LTP pulses before reaching saturation and a similarly sharp decrease during the early LTD cycles. It is important to note that in devices utilizing PVDF-HFP, where multiconductance states are primarily governed by ionic motion, factors such as the semiconductor film morphology and backbone conformation strongly influence charge trapping and detrapping dynamics. These aspects are discussed in more detail below.

The synaptic weight updates are typically modeled by nonlinear equations that describe the conductance during LTP and LTD. This can be better visualized by plotting the normalized conductance as a function of the normalized pulses. Figure 5a shows a representative normalized conductance plot for the PyTr-BT-TT FET. The equations governing the change in conductance during LTP and LTD (*G*_LTP_ and *G*_LTD_, respectively) are shown in the figure, where *G*_max_ and *G*_min_ represent the maximum and minimum conductance, respectively, and *G*_0_ denotes the reference conducting state. The nonlinear parameters during potentiation and depression with a *p* number of pulses are represented by β_p_ and β_d_, respectively. The β values are equal to 1 for an ideal ferroelectric FET, when the amount of the weight increase/decrease during LTP/LTD is linear.

**Figure 5:**
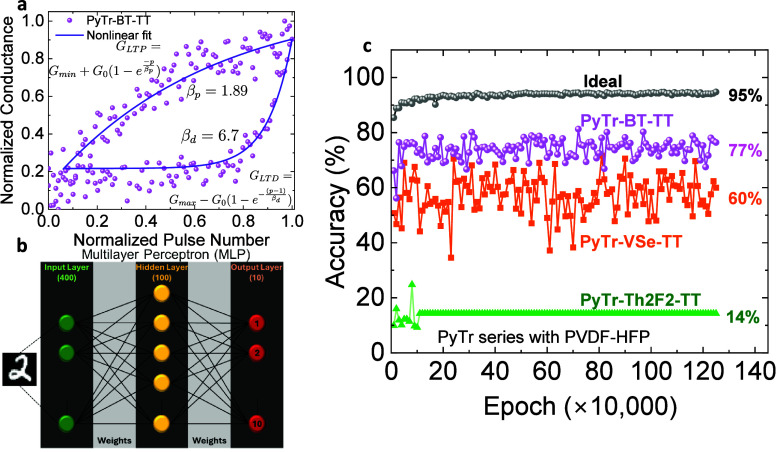
(a) Normalized conductance as a function of the normalized pulse number for a PyTr-BT-TT/PVDF-HFP FET. The scattered symbols are the experimental data, and the bold lines fit two nonlinear equations during LTP and LTD as shown. (b) Representation of the MLP based neural network. (c) Pattern recognition accuracy rate per epoch for the three PyTr series copolymer-based FETs. The gray symbols represent the accuracy rate from an ideal ferroelectric FET.

The synaptic responses of the individual FETs were benchmarked using NeuroSim.^42^ The main input parameters are β_p_, β_d_, and the dynamic range, which depends on the ratio of *G*_max_ and *G*_min_. Specifically, a two-layer MLP (multilayer perceptron) neural network model is implemental for emulating online learning and offline classification using the MNIST handwritten data set. As shown in Figure 5b, the MLP uses an input layer of 400 neurons (20 × 20 images of handwritten digits), 100 hidden layer neurons, and 10 output neurons. In each epoch, the neural network is trained on 10,000 images. The image recognition accuracies for the three PyTr-based FETs are shown in Figure 5c for 125 (×10,000) training epochs. It is not surprising that the PyTr-Th2F2-TT FET yields poor accuracy as the device does not show any synaptic characteristics (Figure 4c). The PyTr-BT-TT FET shows the highest accuracy, reaching ∼80%, followed by the Py-Tr-VSe-TT FET. It should be noted that almost 10 devices were tested for each of the copolymers and the values shown in Figure 5c are typical of all devices. An ideal ferroelectric FET simulated (with no nonlinearity) shows close to 95% accuracy. Along with the nonlinearity parameters, the dynamic range of the conductance plays a role in dictating the accuracies.^29^

Comparing other donor–acceptor copolymers in FET architectures with PVDF-HFP or PVDF-TrFE and without any modification to the semiconductor–dielectric interface,^29^ PyTr-BT-TT shows the best synaptic response and the highest image recognition accuracy rate in a two-layer MLP neural network. We note that higher accuracies, upward of 80%, with electrical and optical synapses have mainly been observed with solution-processable ferroelectric FETs using oxide semiconductors and/or by modifying the interface with nanoparticles.^20,43–45^ In order to understand the differences in the performance of the PyTr synaptic FETs, we look more closely at the interface trap states using both current–voltage characteristics from FETs and capacitance/conductance versus voltage characteristics from metal–insulator–semiconductor (MIS) diodes. In prior work, the complex admittance of PVDF-HFP using metal–insulator–metal capacitors at high frequencies was measured. Compared with other polymer ferroelectric dielectrics, PVDF-HFP showed a lower effective resistance, consistent with ion transport.^29^ Considering that ion transport plays a role in PVDF-HFP–semiconductor interfaces, both the morphology of the films, which is dictated by the linker unit between the donor and the acceptor unit, and the trap states play a role in the synaptic behavior. The shallow trap density of states (DOS), which is typically a Gaussian distribution for polymeric semiconductors, was estimated using the Grünewald’s method;^46^ details are provided in the Supporting Information. Additionally, the interface trap density (*D*_it_) was obtained from capacitance and conductance versus voltage measurements from two-terminal MIS diodes.^47^

### Trap Densities, Morphology, and Structural Properties

Figure 6a shows the shallow trap DOS from the three PyTr-based FETs as a function of energy (starting from the HOMO (highest molecular orbital) level and moving deeper into the band). The analysis was conducted from the linear region of the output characteristics. The semiconductor–dielectric interface potential is shown in Figure S21. All three copolymers have similar trap DOS near the band edge, but moving away from the band edge, the trap DOS of the PyTr-Th2F2-TT FET is at least a few orders of magnitude smaller compared with PyTr-BT-TT and PyTr-VSe-TT.

**Figure 6:**
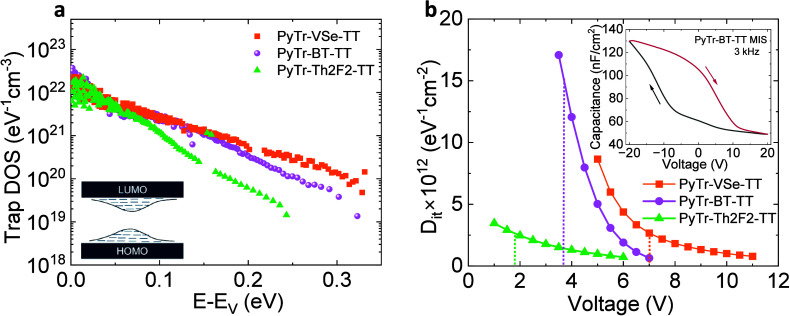
(a) Trap DOS in PyTr-based PVDF-HFP FETs. The inset illustrates the shallow donor-like and acceptor-like trap states. (b) Interface trap density (*D*_it_) obtained from PyTr-based MIS diodes. The inset shows typical hysteresis in the *C*–*V* curve for a PyTr-BT-TT MIS diode. The dashed vertical lines depict the flatband voltage.

The inset of Figure 6b shows a representative capacitance versus voltage (*C*–*V*) hysteresis for the PyTr-BT-TT MIS diode. PyTr-Th2F2-TT and PyTr-VSe-TT diodes show a similar hysteresis in their *C*–*V* curves. The clockwise loop in the *C*–*V* hysteresis of p-type organic MIS diodes is common when polar dielectrics that are used as a high positive voltage may be required to deplete the charges.^23^ Both *C*–*V* and conductance versus voltage (*G*–*V*) measurements for selected frequencies were measured from the PyTr MIS diodes. As the occupancy of the interface trap states changes with gate bias, capacitance and conductance methods from MIS diodes allow the determination of the *D*_it_ values from the loss. Details are provided in the Supporting Information. A clear loss peak, which shifts to lower voltages with an increase in frequency, in the measured conductance is an indication that the interface traps play a role in charge generation and recombination (Figure S22).^48,49^ The *D*_it_ values obtained from the transition region of the *C*–*V* curves for the three MIS devices are shown in Figure 6b. Overall, PyTr-BT-TT has the highest *D*_it_ values. The transition region had a slightly different range for the three MIS diodes with flatband voltages (obtained from the intercept of (*C*_*i*_/*C*)^2^ versus gate bias) being 7, 3.7, and 1.9 V for PyTr-VSe-TT, PyTr-BT-TT, and PyTr-Th2F2-TT, respectively. The *D*_it_ values at the respective flatband voltages are obtained as 3 × 10^12^ eV^–1^ cm^–2^ (PyTr-VSe-TT), 15 × 10^12^ eV^–1^ cm^–2^ (PyTr-BT-TT), and 2.6 × 10^12^ eV^–1^ cm^–2^ (PyTr- Th2F2-TT).

The trap DOS from current–voltage measurements in FETs and the *D*_it_ values from MIS diodes show the same trend. PyTr-Th2F2-TT with PVDF-HFP shows the lowest trap density. Based on this insight, one can reconcile with the synaptic response. Since the multiconductance states arise mainly from ions in PVDF-HFP,^29^ traps in the semiconductor as well as interface trap states play a role in trapping and release of charges, which is central to the synaptic weight update. PyTr-Th2F2-TT with the lowest trap density shows no synaptic response. However, if the multiconductance states arise primarily from switching of domains in the ferroelectric layer, then the trap states play less of a role. As a proof of concept, we fabricated PyTr-Th2F2-TT FETs with PVDF-TrFE, where the ferroelectric domains play a role. These devices demonstrate an increase in synaptic weight during LTP and a decrease in LTD with excellent linearity (Figure S23). However, since these devices were not optimized, the dynamic range was quite low and resulted in a low image recognition accuracy rate.

In addition to the differences in the trap states between the PyTr-based copolymers, their morphology also seems to play a role. The fluorine atoms in donor–acceptor copolymers typically result in molecular aggregation through electronic and noncovalent interactions due to its high electronegativity.^50,51^ Our results from the synaptic devices thus suggest higher aggregation in PyTr-Th2F2-TT, restricting ionic motion. The current–voltage hysteresis in PyTr-Th2F2-TT FETs is minimal compared to PyTr-VSe-TT and PyTr-BT-TT FETs (Figure S19). The atomic force microscope (AFM) images in Figure 7a–c, over the 50 μm × 50 μm scan area, show distinct differences, especially between PyTr-Th2F2-TT and the other two copolymers. The root-mean-square (RMS) roughnesses of PyTr-VSe-TT and PyTr-BT-TT are similarly ∼7.5 ± 1 nm, whereas it is 5.6 ± 0.8 nm in PyTr-Th2F2-TT. While both PyTr-VSe-TT and PyTr-BT-TT show domains in the μm length scales, PyTr-Th2F2-TT shows aggregation at a much smaller length scale.

**Figure 7:**
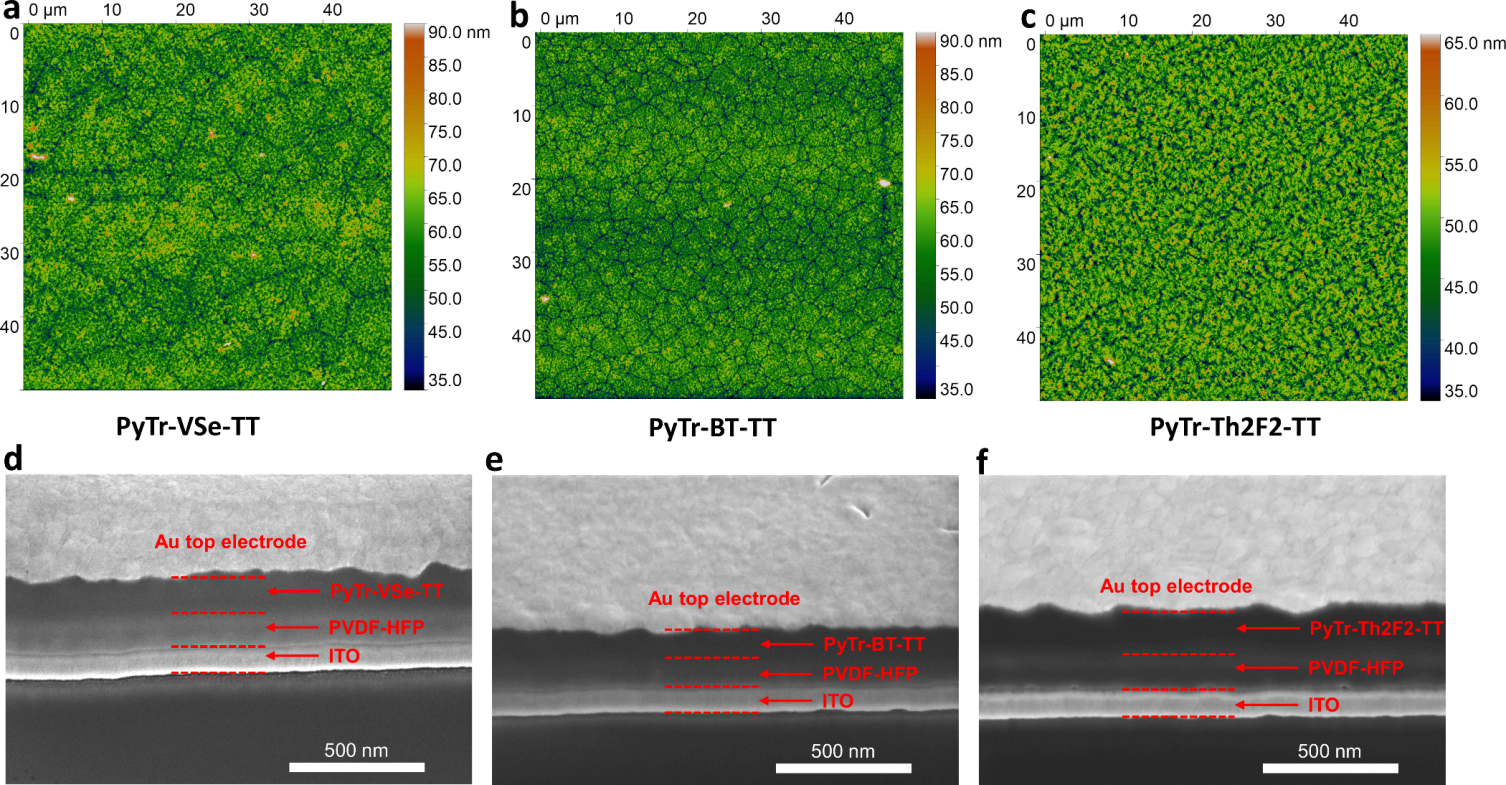
Topographic AFM images from (a) PyTr-VSe-TT, (b) PyTr-BT-TT, and (c) PyTr-Th2F2-TT films grown on PVDF-HFP over a 50 μm × 50 μm scan area. The false color vertical scale is shown on the right of each image. Cross-sectional SEM images of (d) PyTr-VSe-TT, (e) PyTr-BT-TT, and (f) PyTr-Th2F2-TT sandwiched between PVDF-HFP and Au.

XRD was carried out from bulk samples as well as thin films deposited on glass (Figure S24). The overall intensity of PyTr-VSe-TT is high due to the heavy Se atom. The 2θ reflections at ∼3.8° correspond to *d*-spacings between 23.09 and 24.79 Å, which are qualitatively close to the average monomer repeat distance of the three copolymers. The *d*-spacings for the second peak (at ∼7.5°) range from 11.68 to 12.67 Å. These likely correspond to the spacing of the hydrocarbon side chains.^52^ We infer from the XRD data that thin-film formation does not change the translational order in any of the copolymers, and the film formation is consistent with a two-dimensional network of randomly oriented polymer molecules, parallel to the plane of the film but crisscross each other randomly with no relationship between how their axes are directed, which gives rise to the main X-ray scattering peak. This signals translational symmetry within each molecule from repeat unit to repeat unit.

Plasma focused ion beam (PFIB) was used to fabricate cross-sectional samples for scanning electron microscope (SEM) imaging. PFIB cross-sectional milling was performed using an Ar^+^ plasma source on polymers deposited on flexible ITO substrates in order to minimize curtaining due to mismatch in mill rates. To prevent ion beam-induced damage to the polymers, the cross section was fabricated on an area of the sample covered by the Au top electrode, the ion beam current was kept below 2 nA, and ion beam exposure was kept to a minimum. Figure 7d–f shows the cross-sectional images. The interface between the PyTr copolymers and PVDF-HFP is visually demarcated by a change in contrast (indicated with dashed red lines in Figure 7d–f in all cases. Additionally, the Au/PyTr copolymer interfaces are similar for all three. These images further confirm that the interface geometries are similar for all three copolymers. The data are consistent with differences observed in the synaptic characteristics for devices made from different copolymers being governed by differences in morphology and trap states as enumerated above and not due to physical differences at the interface.

## Conclusions

In summary, this work highlights the critical role of the dielectric (ferroelectric)–organic semiconductor interface in FET architectures for synaptic responses (LTP/LTD). Using the relaxor ferroelectric dielectric, PVDF-HFP, where multiconductance states are primarily ion-driven, together with three PyTr acceptor-based copolymers featuring distinct linker units, we demonstrate that the LTP/LTD behavior strongly depends on interface trap densities and the morphology of the PyTr copolymer films. While all three copolymers—PyTr-VSe-TT, PyTr-BT-TT, and PyTr-Th2F2-TT—exhibit similar FET performance with p*-*type transport and operating voltages below 8 V, their synaptic responses differ significantly. Among them, PyTr-BT-TT shows the most efficient synaptic weight update during LTP and LTD, followed by PyTr-VSe-TT, whereas PyTr-Th2F2-TT displays negligible synaptic modulation.

The synaptic devices were further evaluated for image recognition using a two-layer MLP network. PyTr-BT-TT achieved the highest accuracy of ∼80%, outperforming not only the other two copolymers but also other polymer FET architectures relying solely on a ferroelectric polymer dielectric and an organic semiconductor. In contrast, PyTr-Th2F2-TT yielded very low recognition accuracy (<20%), consistent with AFM images revealing submicrometer aggregates likely induced by fluorine atoms. Detailed trap DOS estimations from FET characteristics, along with *D*_it_ measurements from MIS diodes, confirm that PyTr-Th2F2-TT exhibits the lowest trap density, further underscoring the importance of charge trapping and release via interface states in governing synaptic weight updates when using PVDF-HFP.

As polymer ferroelectrics continue to gain traction in organic neuromorphic devices, it is clear that when ions dominate the multiconductance states, as in PVDF-HFP, the film morphology and interface trap densities play a decisive role in synaptic performance. Organic semiconductors from the same family may exhibit similar transistor characteristics yet behave very differently as neuromorphic devices due to subtle structural variations. This study highlights opportunities to further enhance neural network image recognition by careful molecular design of donor–acceptor copolymers for synaptic transistors.

## Experimental Methods and Analysis

### Materials

Poly­(vinylidene fluoride-*co*-hexafluoropropylene) (PVDF-HFP) with molecular weight (Mw) = 455,000 was obtained from Sigma-Aldrich. *N*,*N*-Dimethylformamide (DMF) and anhydrous 1,2-dichlorobenzene (98%) were also purchased from Sigma-Aldrich (St. Louis, MO, USA). The synthesis and characterization of the donor–acceptor copolymers PyTr-VSe-TT, PyTr-BT-TT, and PyTr-Th2F2-TT are discussed in the Supporting Information.

### Device Fabrication

#### Field-Effect Transistors

Glass substrates (1″ × 1″) were cleaned through an organic cleaning process and subsequently coated with a 50 nm Al gate electrode via thermal evaporation through a patterned shadow mask. The ferroelectric dielectric layer was prepared by dissolving PVDF-HFP in DMF (100 mg mL^–1^). The solution was heated at 80 °C for 3 h and stirred overnight at room temperature. To obtain thinner dielectric films, the solution was diluted to 50 mg mL^–1^ prior to spin-coating. Under a nitrogen atmosphere, the solution was statically dispensed onto the Al gate and spin-coated at 1600 rpm for 60 s. The coated substrates were then annealed at 70 °C for 10 min in nitrogen to remove residual solvent, resulting in a dielectric thickness of approximately 50 nm.

Semiconducting layers of PyTr-VSe-TT, PyTr-BT-TT, and PyTr-Th2F2-TT were prepared by dissolving each polymer in 1,2-dichlorobenzene (5 mg mL^–1^). The solutions were sequentially heated at 100 °C for 1 h, 130 °C for 1 h, and 145 °C for over 12 h while stirring at 200 rpm. After cooling and overnight stirring at room temperature, the solutions were filtered through a 0.45 μm PTFE filter and reheated at 145 °C for 30 min prior to spin-coating. Under nitrogen, 75 μL of solution was dynamically spin-coated onto the ferroelectric layer at 900 rpm for 60 s. To restrict film coverage to the channel region, Teflon tape was applied during spin-coating and removed prior to annealing at 120 °C for 1 h in nitrogen.

Source and drain electrodes were formed by thermally evaporating a 50 nm Au layer through a patterned mask. Each substrate contained four devices with a fixed channel width of 1000 μm and channel lengths of 50, 75, 100, and 125 μm.

#### Metal–Insulator–Semiconductor Diodes (MIS)

For MIS capacitors, a 50 nm Al gate was uniformly deposited on precleaned glass substrates by thermal evaporation. The PVDF-HFP dielectric layer was spin-coated using the same procedure as described above. Semiconductor copolymers were then spin-coated onto the dielectric layer following the same protocol as in device fabrication. Finally, Au pads (50 nm) were thermally evaporated through a patterned shadow mask to define capacitance structures. Each substrate contained circular pads with diameters of 250, 500, 750, and 1000 μm, which were used for capacitance measurements.

### Characterization

#### Electrical Measurements

Current–voltage characteristics were measured at room temperature using a Keithley 4200A-SCS parameter analyzer. Capacitance and conductance–voltage measurements were performed with an HP 4284A LCR meter. The dynamic current measurements were performed with a lock-in amplifier from Zurich Instruments (MFLI). The currents measured by the MFLI were scaled with respect to the offset and the gain factor to estimate EPSC and IPSC. As a check, the zero gate bias values were consistent with the values obtained by a Keithley 4200A. A Python script was used to estimate the average value of EPSC/IPSC, which lasted over 1 s cycles, and dividing it by the drain-source voltage resulted in the conductance values as a function of the pulse number.

#### Atomic Force Microscopy (AFM)

The surface morphology of the polymer films on PVDF-HFP was analyzed with a Bruker Innova AFM operated in tapping mode.

#### Scanning Electron Microscopy (SEM)

SEM characterization was performed using a Thermo Fisher Helios Hydra UX DualBeam PFIB/SEM. A cross-sectional trench with 10 μm width × 2 μm length × 2 μm depth was milled from an area of the sample with a Au top electrode using an Ar^+^ plasma ion source at a 30 kV accelerating voltage and a 2 nA beam current. The trench was kept relatively shallow to minimize ion beam exposure to the organic layers, which are beam-sensitive. SEM imaging was performed in immersion mode using the TLD detector at a 2 kV accelerating voltage and a 50 pA beam current.

#### X-ray Diffraction (XRD)

Powder X-ray diffraction was measured on a Rigaku Miniflex diffractometer using Ni-filtered Cu Kα radiation. Samples of either bulk polymer materials or films deposited on glass microscope coverslips as substrates were affixed to the surface of a proprietary low-background silicon sample holder using high-vacuum grease. Samples were analyzed in Bragg–Brentano geometry using continuous θ/2θ scans with a step size of 0.02°. SmartLab Studio II, version 4.6.671.0, Rigaku Corporation, Tokyo, Japan (2014), was used for data collection.

### Transistor Parameters

The drain current (*I*_D_) in the saturation region is given by *I*_D_(sat) = (*W*/2*L*)­μ*C*_*i*_(*V*_GS_ – *V*_th_)^2^, where *W* and *L* are the channel width and length, respectively. *C*_*i*_ is the capacitance/area of the dielectric, *V*_GS_ is the gate-source voltage, and *V*_th_ is the threshold voltage. The carrier mobility in the saturation region was extracted from $\mu_{\mathrm{sat}}=\left(2L/WC_{i}\right){\left(\partial \sqrt{I_{D}}/\partial V_{\mathrm{GS}}\right)}^{2}$ . The subthreshold swing is given by SS = d*V*_GS_/(log *I*_D_).

## Supplementary Material



## Notes

Synthesis protocols of PyTr copolymers, NMR characterization, GPC, DSC, current–voltage characteristics of FETs, synaptic performance of PyTr-BT-TT FETs, trap DOS analysis from FETs, interface trap density from capacitance/conductance measurements, normalized conductance from a PyTr-Th2F2-TT/PVDF-TrFE FET, and XRD (PDF)
