# Supplementary material for: Structure–Function Coupling in Pyridyl Triazole Copolymers for Neuromorphic Synaptic Transistors
Source: ACS Appl Electron Mater. 2026 Feb 12;8(6):2408–19. doi: 10.1021/acsaelm.5c02633 (PMC13019670; doi:10.1021/acsaelm.5c02633)
Supplement: Supplementary file 1 [file el5c02633_si_001.pdf]

Supporting Information

Structure–Function Coupling in Pyridyl-Triazole Copolymers for Neuromorphic Synaptic Transistors

Arash Ghobadi[1], Salahuddin Attar[2], Abhijeet Abhi[1], Thomas B. Kallaos[1], Dilan M. Gamachchi[1], Indeewari M. Karunarathne[1], Andrew C. Meng[1, 3], Joseph C. Mathai[3, 4], Shubhra Gangopadhyay[4], Steven P. Kelley[5], Mohammed Al-Hashimi[2*], and Suchismita Guha[1, 3*]

[1] Department of Physics and Astronomy, University of Missouri, Columbia, MO 65211, USA

[2] College of Science and Engineering, Hamad Bin Khalifa University, Doha 34110, Qatar

[3] MU Materials Science and Engineering Institute, University of Missouri, Columbia, MO 65211, USA

[4] Department of Electrical Engineering and Computer Science, University of Missouri, Columbia, MO 65211, USA

[5] Department of Chemistry, University of Missouri, Columbia, MO 65211, USA

[*] Corresponding authors E-mail: guhas@missouri.edu, malhashimi@hbku.edu.qa

Contents

1. Materials and Characterization 3

2. Synthetic Procedures 4

2.1 Synthesis of 4,7-dibromo-2H-[1,2,3]triazolo[4,5-c]pyridine (2) 4

2.2 Synthesis of 4,7-dibromo-2-(4-decyltetradecyl)-2H-triazolo [4,5-c]pyridine (4) 4

2.3 Synthesis of (E)-1,2-bis(5-(7-bromo-2-(4-decylhexadecyl)-2H-[1,2,3]triazolo[4,5-c]pyridin-4-yl)selenophen-2-yl)ethene (M1) 5

2.4 Synthesis of 4,7-bis(5-(7-bromo-2-(4-decylhexadecyl)-2H-[1,2,3]triazolo[4,5-c]pyridin-4-yl)thiophen-2-yl)benzo[c][1,2,5]thiadiazole (M2) 6

2.5 Synthesis of 4,4'-(3,3'-difluoro-[2,2'-bithiophene]-5,5'-diyl)bis(7-bromo-2-(4-decylhexadecyl)-2H-[1,2,3]triazolo[4,5-c]pyridine) (M3) 6

2.5 Synthesis of Copolymers (PyTr-VSe-TT, PyTr-BT-TT, and PyTr-Th2F2-TT) 7

3. NMR Spectroscopic Characterization 9

3.1 ^{1}H NMR spectra of intermediate 4 9

3.2 ^{13}C NMR spectra of intermediate 4 9

3.3 ^{1}H NMR spectra of monomer M1 10

3.4 ^{13}C NMR spectra of monomer M1 10

3.5 ^{1}H NMR spectra of monomer M2 11

3.6 ^{13}C NMR spectra of monomer M2 11

3.7 ^{1}H NMR spectra of monomer M3 12

3.8 ^{13}C NMR spectra of monomer M3 12

3.9 ^{19}FNMR spectra of monomer M3 13

3.10 ^{1}H NMR spectra of Polymer PyTr-VSe-TT 13

3.11 ^{1}H NMR spectra of Polymer PyTr-BT-TT 14

3.12 ^{1}H NMR spectra of Polymer PyTr-Th2F2-TT 14

4. Gel Permeation Chromatography 15

4.1 GPC of PyTr-VSe-TT 15

4.2 GPC of PyTr-BT-TT 16

4.3 GPC of PyTr-Th2F2-TT 17

5. Thermal Studies 18

5.1 TGA analysis 18

5.2 DSC analysis 18

6. Transistor Characteristics 19

7. Synaptic Characteristics 20

8. Trap Density of States Analysis 20
9. Interface Trap Density (Dit) from Capacitance and Conductance Measurements 21
10. Normalized Conductance from PyTr-Th2F2-TT/PVDF-TrFE FET 22
11. X-ray Diffraction 23
12. References 23

1. Materials and Characterization

All commercially available solvents were distilled and freshly dried by standard drying methods. Reagents and chemicals were used as received without further purification unless otherwise stated. All the stannylated monomers were purchased from Solarmer inc. Unless otherwise stated, all operations and reactions were carried out under argon using standard Schlenk line techniques. Analytical thin-layer chromatography was performed on Merck aluminum-backed plates pre-coated with silica (0.2 mm, 60 F254) gel. Plates were visualized by exposure to UV light (254 nm) or (365 nm). ^{1}H and ^{13}C NMR spectra were recorded on a Bruker AV-400 (400 MHz), using the residual solvent resonance of $CDCl_3$ or TMS as an internal reference and are given in ppm. Number-average (Mn) and weight average (Mw) were determined by Agilent Technologies 1200 series GPC running in chlorobenzene at 80 °C, using two PL mixed B columns in series, and calibrated against narrow polydispersity polystyrene standards. UV-vis spectra were recorded on a PerkinElmer Lambda 1050 UV-Vis spectrometer. Differential scanning calorimetry (DSC) analysis were recorded on PerkinElmer Jade DSC Differential Scanning Calorimeter under nitrogen at 5 °C min-1 of heating rate from 30 °C to 350 °C in two cycles heating-cooling, and thermogravimetric analysis (TGA) curves were collected on Mettler in nitrogen at 10 0C min-1 of heating rate from 30 °C to 600 °C. Cyclic voltammetry (CV) measurements of polymers films were performed under argon atmosphere using a CHI760E Voltammetry analyzer with 0.1 M tetra-n-butylammonium hexafluorophosphate in acetonitrile as the supporting electrolyte. A glassy carbon working electrode, a platinum wire counter electrode, and a silver wire ($Ag/AgNO_3$) reference electrode were employed, and the ferrocene/ferrocenium (Fc/Fc+) was used as the internal reference for all measurements. The scanning rate was 100 mV/s. Polymer films were drop-casted from chloroform solutions on a glassy carbon working electrode (2 mm in diameter).

The monomers of PyTr-VSe-TT, PyTr-BT-TT, and PyTr-Th2F2-TT are referred to as **M1**, **M2**, and **M3**, respectively.

2. Synthetic Procedures

2.1 Synthesis of 4,7-dibromo-2H-[1,2,3]triazolo[4,5-c]pyridine (2)

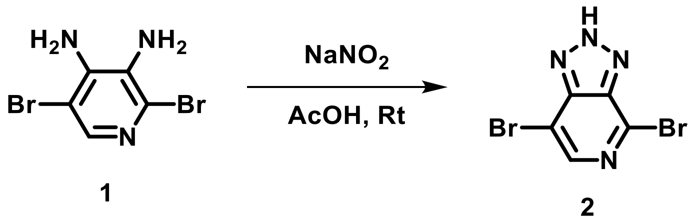

Scheme S1: Synthesis of 4,7-dibromo-2H-[1,2,3]triazolo[4,5-c]pyridine (2).

To a stirred solution of 2,5-dibromo-3,4-diaminopyridine (2.00 g, 7.49 mmol) in acetic acid (30 ml) was added dropwise a solution of NaNO2 (0.775 g, 11.23 mmol) in 10 mL distilled water. The mixture was stirred at room temperature for 24 h. The solid precipitation was filtered, washed with distilled water and then isopropanol to obtain a white solid (2.1 g, 98.1%). The product (2) (2.1 g, 98.1%) obtained after drying under vacuum was directly used for the next step without further purification.

2.2 Synthesis of 4,7-dibromo-2-(4-decyltetradecyl)-2H-triazolo [4,5-c]pyridine (4)

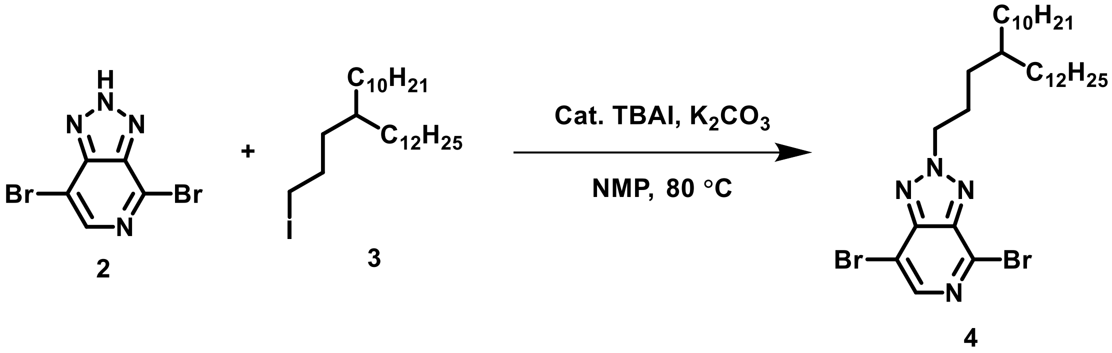

Scheme S2: Synthesis of 4,7-dibromo-2-(4-decyltetradecyl)-2H-triazolo [4,5-c]pyridine (4).

To a stirred solution of 4,7-dibromo-2H-[1,2,3]triazolo[4,5-c]pyridine (2.00 g, 7.20 mmol) in N-methyl-2-pyrrolidone (25 mL) under argon atmosphere was added K_2CO_3 (1.99 g, 14.40 mmol). The mixture was stirred at 80 °C for 30 min. 11-(3-iodopropyl)tricosane (4.13 g, 8.64 mmol) and tetrabutylammonium Iodide (0.266 g, 0.72 mmol) was added dropwise to the mixture, and then stirred at 80 °C for 24 h under

argon. The reaction mixture was added water (100 mL) The solution was extracted with Ether (Et_2O) and then washed with water and brine, and dried over with $MgSO_4$. After concentration under reduced pressure, the crude product was purified by silica gel chromatography with eluent (10 % $CHCl_3$:Hexane) to obtain a colorless liquid (4.21 g, 95.2%). ^{1}H NMR (400 MHz, $CDCl_3$) δ 8.34 (s, 1H), 4.80 (t, J = 7.4 Hz, 2H), 2.25 – 2.04 (m, 2H), 1.48 – 1.10 (m, 43H), 0.88 (t, J = 6.8 Hz, 6H). ^{13}C NMR (101 MHz, $CDCl_3$) δ 146.31, 143.17, 141.58, 132.62, 108.02, 58.38, 36.89, 33.37, 31.90, 30.13, 30.01, 29.66, 29.63, 29.34, 27.27, 26.58, 22.67, 14.10.

2.3 Synthesis of (E)-1,2-bis(5-(7-bromo-2-(4-decylhexadecyl)-2H-[1,2,3]triazolo[4,5-c]pyridin-4-yl)selenophen-2-yl)ethene (M1)

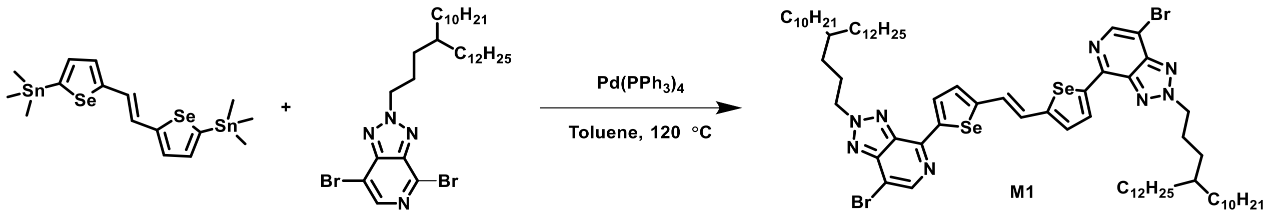

Scheme S3: Synthesis of monomer (M1).

The headspace of sealed microwave vial containing 3 (0.5g, 0.778 mmol), (E)-1,2-bis(5-(trimethylstannyl)thiophen-2-yl)ethene (0.238 g, 0.389 mmol) and Tetrakis(triphenylphosphine)palladium(0) (22.4 mg, 0.019 mmol) was deoxygenated using Argon flow. Anhydrous and degassed toluene (3 ml) was injected in reaction mixture and then heated to 120 ℃ for 15 h. The reaction mixture was filtered through a celite-silica plug and concentrated under vacuum to obtain crude solid. The product was further stirred in methanol (50 mL), filtered, dried to obtain orange-red solid (0.46 g, 83.9 %).

^{1}H NMR (400 MHz, $CDCl_3$) δ 8.46 (d, J = 4.0 Hz, 2H), 8.33 (s, 2H), 7.25 (d, J = 4.1 Hz, 2H), 7.02 (s, 2H), 4.72 (t, J = 7.2 Hz, 4H), 2.23 – 1.94 (m, 4H), 1.47 – 0.91 (m, 86H), 0.81-0.78 (m, 12H). ^{13}C NMR (101 MHz, $CDCl_3$) δ 151.89, 146.97, 146.54, 145.58, 143.72, 137.92, 133.88, 131.76, 126.91, 104.99, 57.98, 36.97, 33.46, 31.95, 30.28, 30.09, 29.73, 29.70, 29.39, 27.33, 26.66, 22.71, 14.14.

2.4 Synthesis of 4,7-bis(5-(7-bromo-2-(4-decylhexadecyl)-2H-[1,2,3]triazolo[4,5-c]pyridin-4-yl)thiophen-2-yl)benzo[c][1,2,5]thiadiazole (M2)

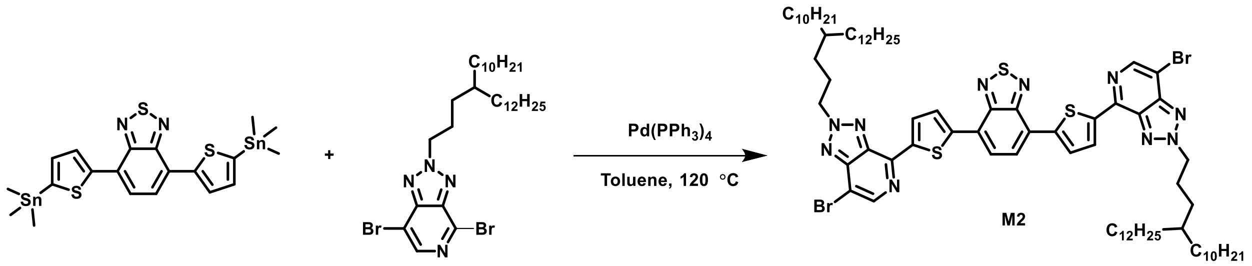

Scheme S4: Synthesis of monomer (M2).

M2 (0.486 g, 87.8 %) was obtained following the procedure for **M1**. ^{1}H NMR (400 MHz, $CDCl_3$) δ 8.35 (s, 2H), 8.23 (d, *J* = 4.0 Hz, 2H), 7.97 (d, *J* = 4.0 Hz, 2H), 7.73 (s, 2H), 4.69 (t, *J* = 7.2 Hz, 4H), 2.09 (s, 4H), 1.49 – 0.92 (m, 86H), 0.78 (t, *J* = 6.6 Hz, 12H). ^{13}C NMR (101 MHz, $CDCl_3$) δ 152.06, 146.75, 145.09, 143.31, 142.65, 141.57, 138.10, 131.33, 128.61, 125.46, 125.37, 104.96, 57.89, 37.03, 33.47, 31.95, 30.39, 30.14, 29.76, 29.70, 29.40, 27.30, 26.69, 22.71, 14.15.

2.5 Synthesis of 4,4'-(3,3'-difluoro-[2,2'-bithiophene]-5,5'-diyl)bis(7-bromo-2-(4-decylhexadecyl)-2H-[1,2,3]triazolo[4,5-c]pyridine) (M3)

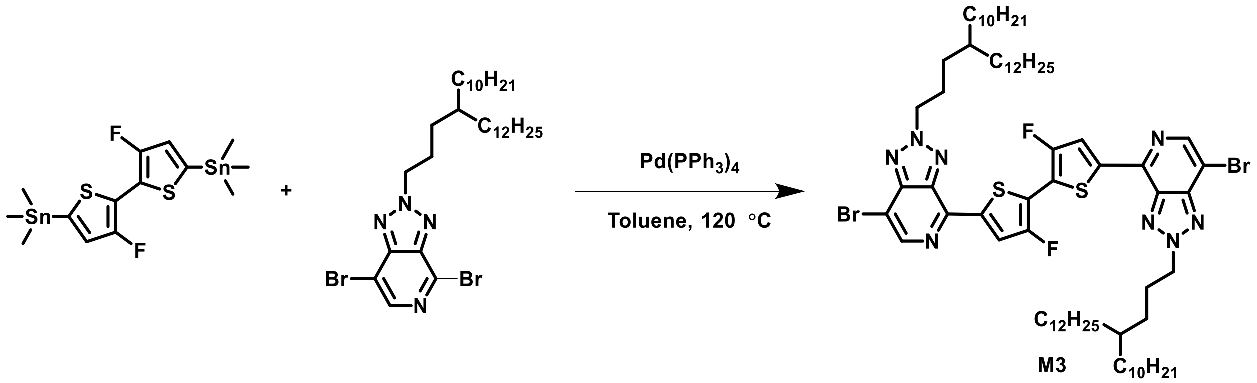

Scheme S5: Synthesis of monomer (M3).

M3 (0.44 g, 85.4 %) was obtained following the procedure for **M1**. ^{1}H NMR (400 MHz, $CDCl_3$) δ 8.26 (s, 2H), 8.03 (s, 2H), 4.71 (t, *J* = 7.2 Hz, 4H), 2.24 – 1.94 (m, 4H), 1.53 – 0.91 (m, 86H), 0.78 (t, *J* = 6.8 Hz, 12H). ^{13}C NMR (101 MHz, $CDCl_3$) δ 155.79 (s), 153.14 (s), 146.86 (s), 143.86 (s), 143.12 (s), 137.92 (s), 137.16 (t,

J = 7.7 Hz), 119.88 (d, *J* = 25.8 Hz), 116.48 – 116.05 (m), 105.95 (s), 58.10 (s), 37.00 (s), 33.46 (s), 31.94 (s), 30.33 (s), 30.10 (s), 29.71 (d, *J* = 4.5 Hz), 29.39 (s), 27.36 (s), 26.67 (s), 22.70 (s), 14.13 (s). ^{19}F NMR (376 MHz, $CDCl_3$) δ -119.20.

2.5 Synthesis of Copolymers (PyTr-VSe-TT, PyTr-BT-TT, and PyTr-Th2F2-TT)

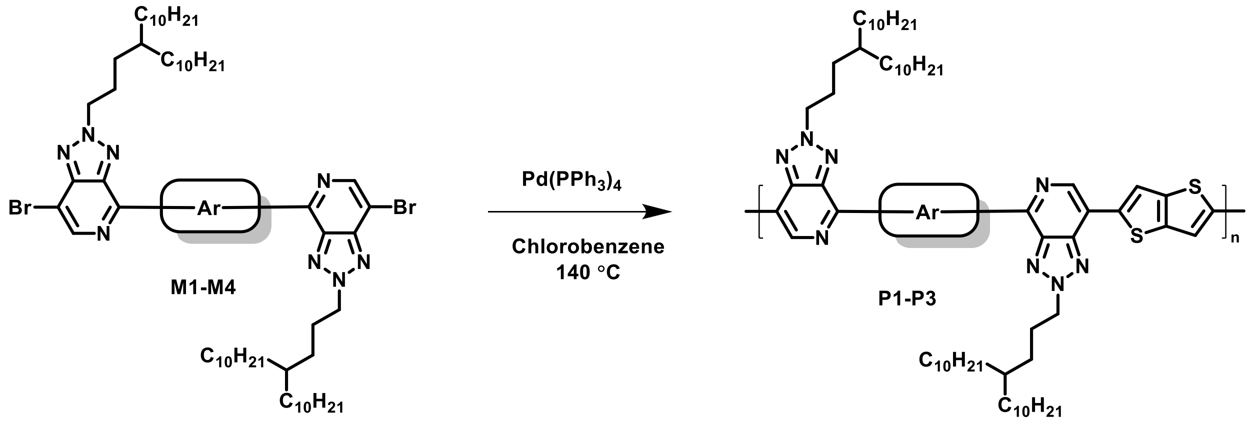

Scheme S6: Synthesis of copolymers.

A 2–5 mL microwave vial was charged with monomer **M1–M4** (1.0 mmol), $Pd(PPh_3)_4$ (0.025 mmol), and the corresponding trimethylstannyl monomer (1.0 mmol). The vial was sealed and purged with argon for 15 minutes. Anhydrous, deoxygenated chlorobenzene (3 mL) was then added, and the mixture was further degassed by bubbling argon for an additional 20 minutes. The sealed vial was placed in a preheated metal block at 140 °C and stirred overnight. After cooling to room temperature, the reaction mixture was poured into acidic methanol (~2% HCl in methanol, 200 mL), resulting in the formation of dark polymer fragments. The precipitated polymers were collected by filtration using a thimble and purified by sequential Soxhlet extraction with methanol, acetone, hexane, and chloroform. The chloroform fraction was concentrated and reprecipitated in methanol to afford the copolymers PyTr-VSe-TT, PyTr-BT-TT, and PyTr-Th2F2-TT in 50–65% yield. The number-average molecular weights (Mn) and polydispersity indices (PDI) of the polymers were determined by high-temperature gel permeation chromatography (GPC) using chlorobenzene as the eluent at 85 °C with a flow rate of 0.8 mL min^{-1}. The measurements were performed

on an Agilent PL-GPC 220 system equipped with PLgel MIXED-B columns (10 µm, 300 × 7.5 mm) and a differential refractive index detector. The system was calibrated with narrow polystyrene standards, and polymer samples were dissolved in chlorobenzene (2 mg mL^{-1}) and filtered through 0.45 µm PTFE syringe filters prior to injection. Under these conditions, the Mn and PDI values were determined to be 59.4 kDa (PDI = 1.5) for P1, 67.0 kDa (PDI = 2.2) for P2, and 224.3 kDa (PDI = 2.7) for PyTr-Th2F2-TT . The relatively higher molecular weight of PyTr-Th2F2-TT can be attributed to its more planar and rigid conjugated backbone due to nonbonding S-F interactions, which promotes effective π–π stacking and enhances chain propagation during polymerization. In contrast, the incorporation of bulkier or twisted aromatic linkers in PyTr-VSe-TT and PyTr-BT-TT may introduce steric hindrance that limits chain growth, resulting in comparatively lower molecular weights. Overall, the observed GPC results indicate that subtle structural modifications within the donor–acceptor backbone have a pronounced influence on polymer chain length, dispersity, and processability.

Table S1. Summary of molecular weights, thermal, optical, electrochemical, parameters of all the polymers.

Polymer	***M_n,*** ***[a]*** ***kDa***	***Đ [b]***	***T_d^{onset} [°C]***	***λ max solution (nm)[d]***	***λ max film (nm)[d]***	***E_g^{opt} [ev][i]***	***LUMO[g] [ev]***	***HOMO[g] [ev]***	***E_g^{ec} [ev][h]***
PyTr-VSe-TT	59	1.5	414	630, 689	624, 684	1.68	-3.06	-5.05	1.99
PyTr-BT-TT	67	2.2	420	628, 678	632, 692	1.62	-3.20	-5.24	2.04
PyTr-Th2F2-TT	224	2.7	436	609, 662	606, 669	1.69	-3.00	-5.30	2.30

3. NMR Spectroscopic Characterization

3.1 ^{1}H NMR spectra of intermediate 4

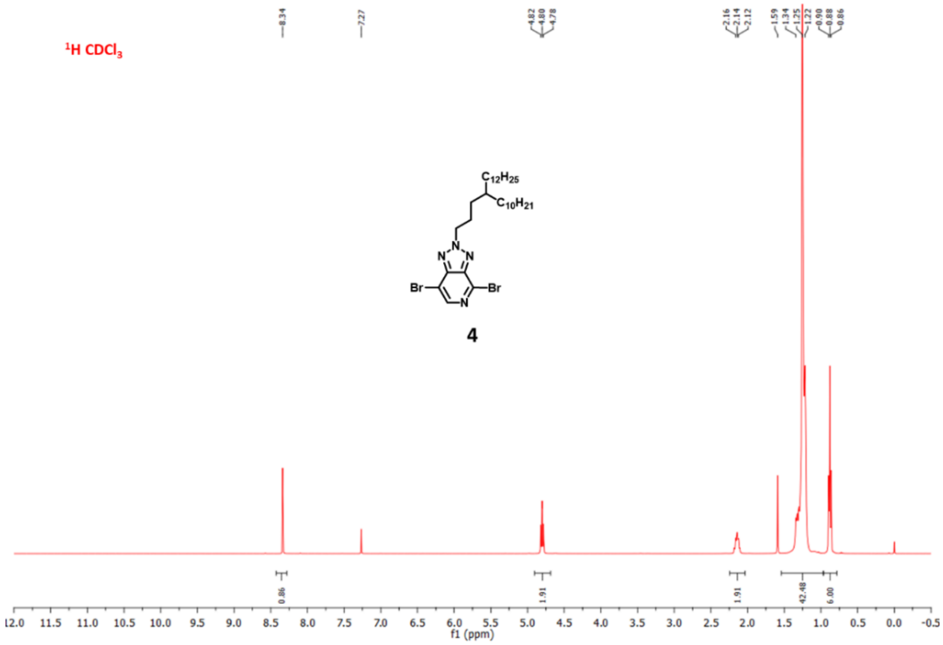

Figure S1: ^{1}H NMR ($CDCl_3$, 298 K, 400 MHz) of intermediate 4.

3.2 ^{13}C NMR spectra of intermediate 4

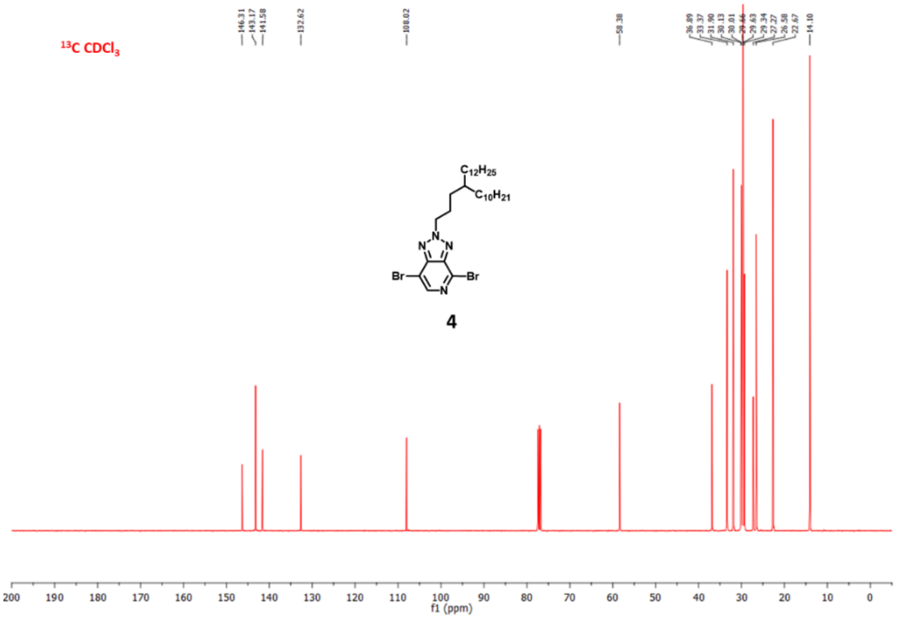

Figure S2: ^{13}C NMR ($CDCl_3$, 298 K, 400 MHz) of intermediate 4.

3.3 ^{1}H NMR spectra of monomer M1

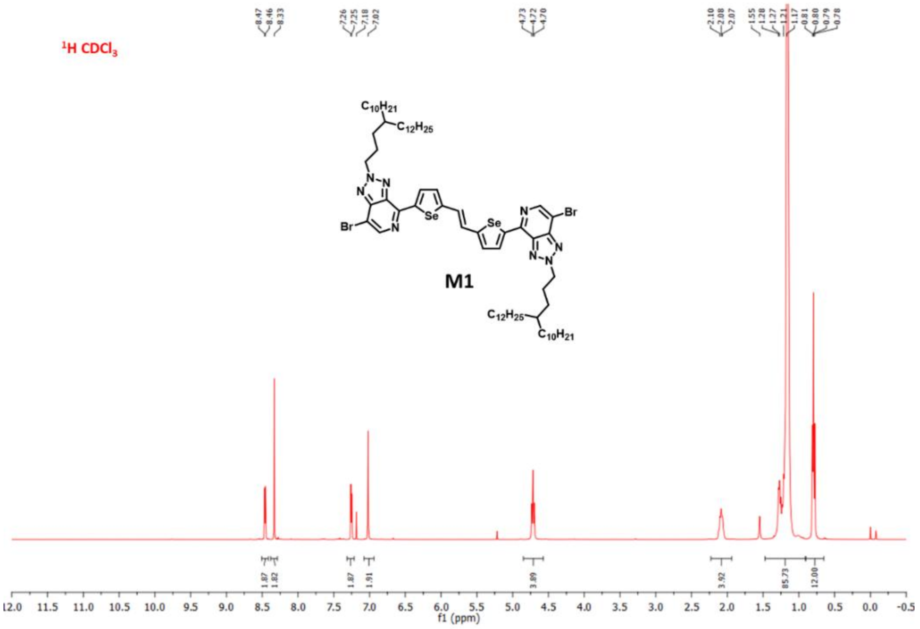

Figure S3: ^{1}H NMR ($CDCl_3$, 298 K, 400 MHz) of M1.

3.4 ^{13}C NMR spectra of monomer M1

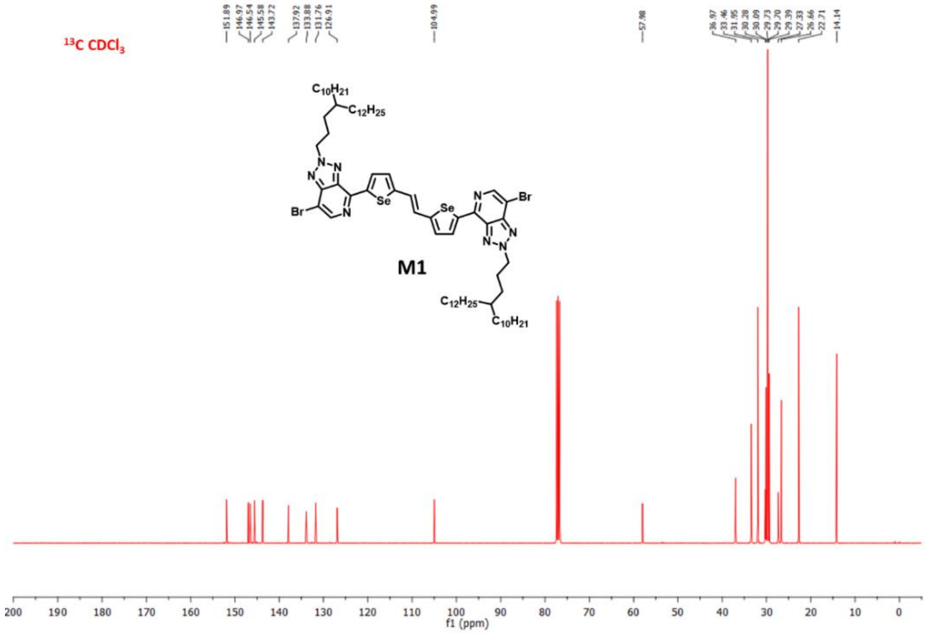

Figure S4: ^{13}C NMR ($CDCl_3$, 298 K, 400 MHz) of M1.

3.5 ^{1}H NMR spectra of monomer M2

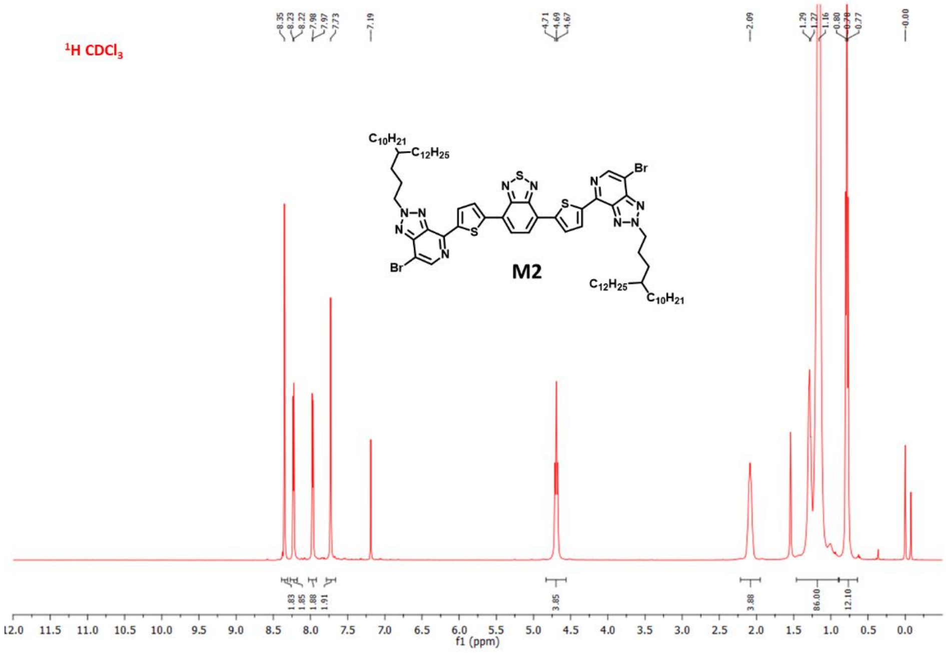

Figure S5: ^{1}H NMR ($CDCl_3$, 298 K, 400 MHz) of M2.

3.6 ^{13}C NMR spectra of monomer M2

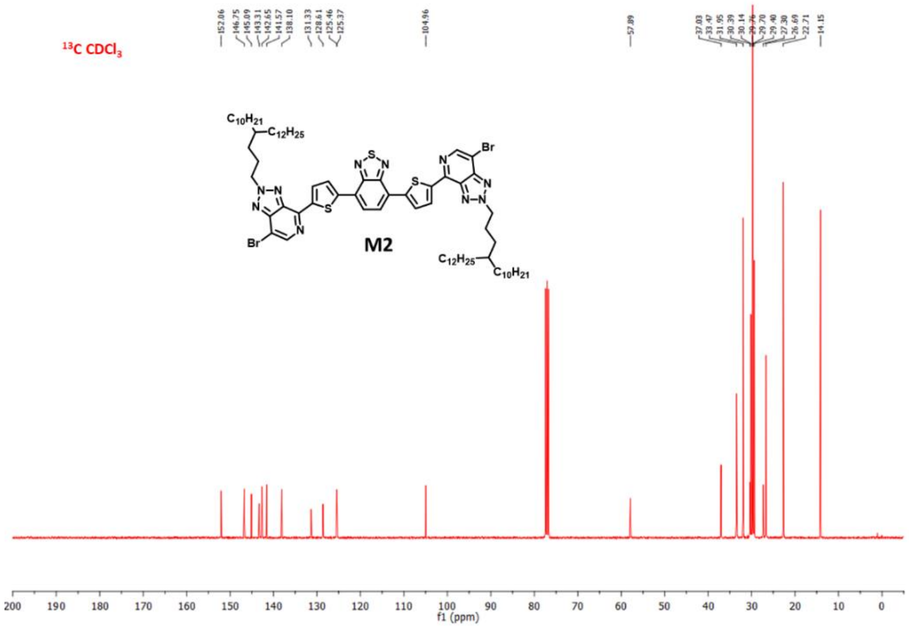

Figure S6: ^{13}C NMR ($CDCl_3$, 298 K, 400 MHz) of M2.

3.7 ^{1}H NMR spectra of monomer M3

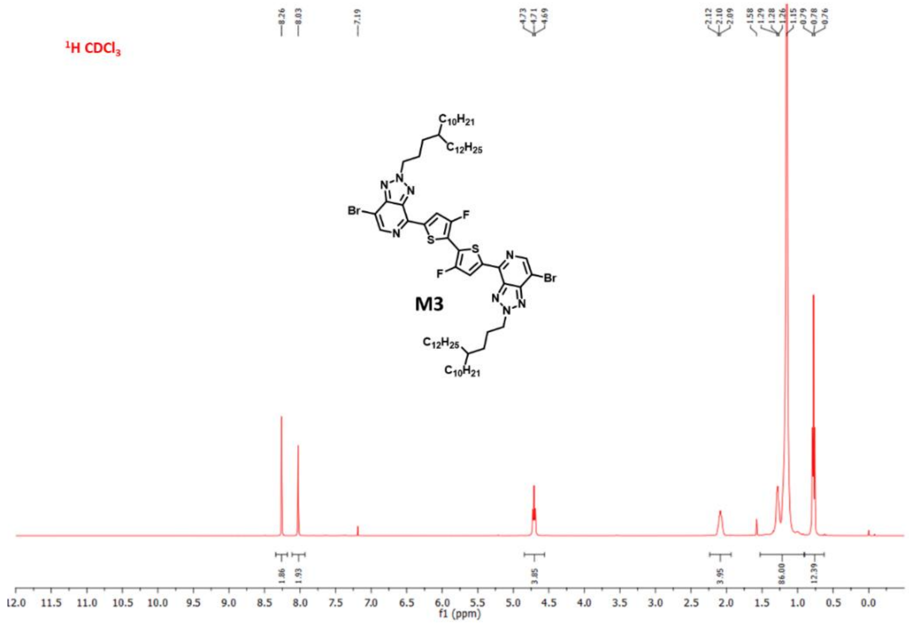

Figure S7: ^{1}H NMR ($CDCl_3$, 298 K, 400 MHz) of M3.

3.8 ^{13}C NMR spectra of monomer M3

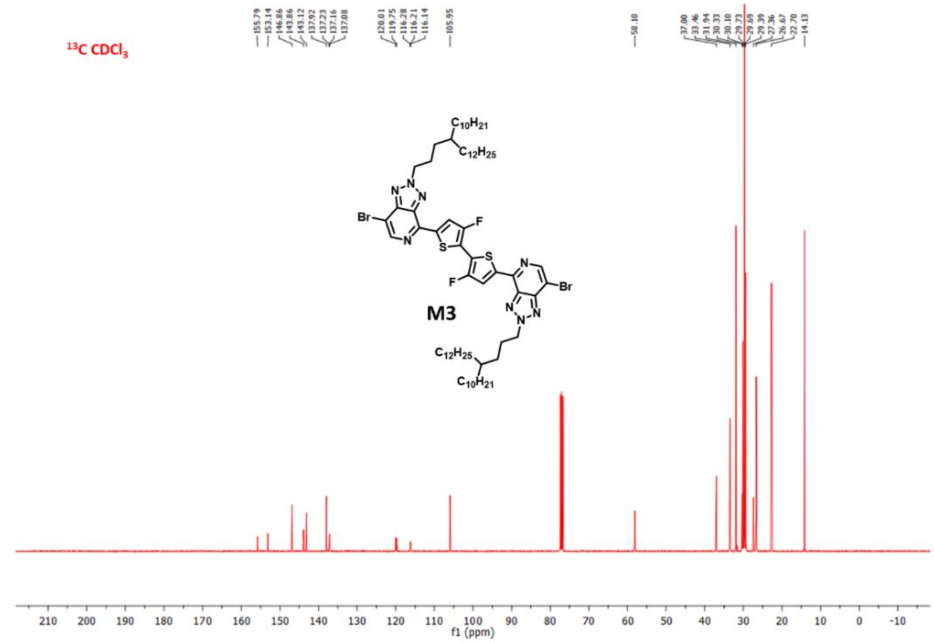

Figure S8: ^{13}C NMR ($CDCl_3$, 298 K, 400 MHz) of M3.

3.9 ^{19}FNMR spectra of monomer M3

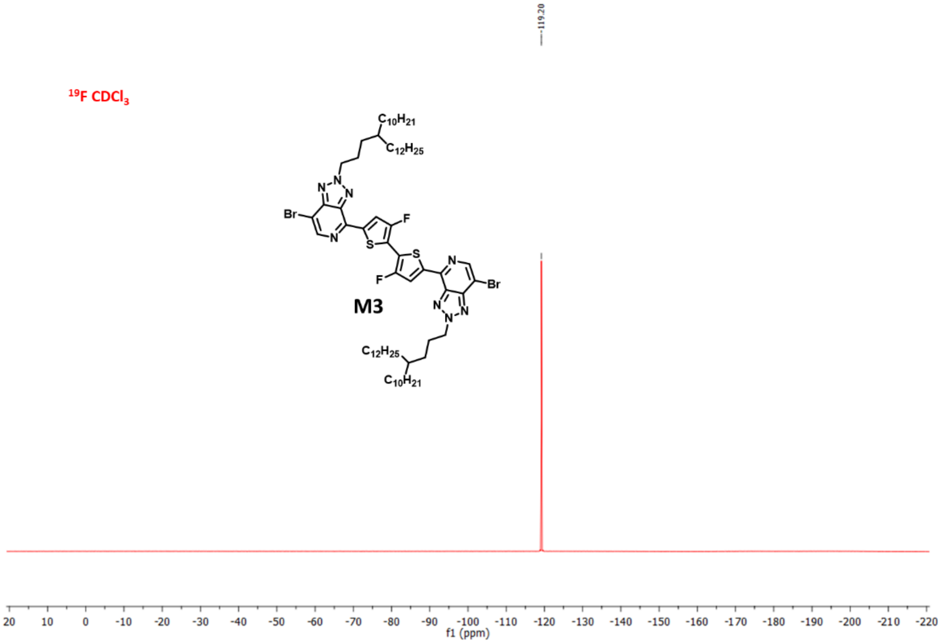

Figure S9: ^{19}FNMR ($CDCl_3$, 298 K, 400 MHz) of M3.

3.10 ^{1}H NMR spectra of Polymer PyTr-VSe-TT

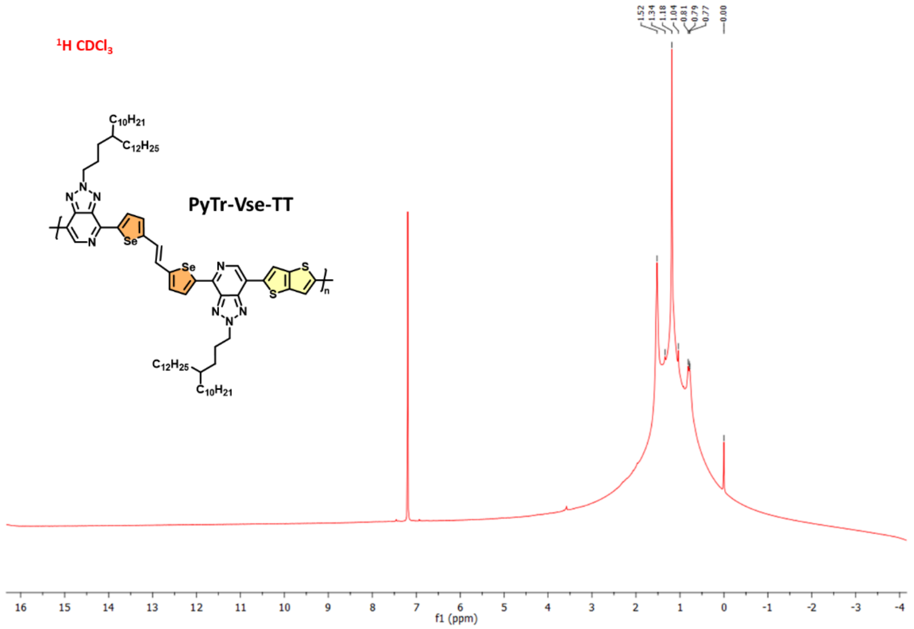

Figure S10: ^{1}H NMR ($CDCl_3$, 298 K, 400 MHz) of PyTr-VSe-TT

3.11 ^{1}H NMR spectra of Polymer PyTr-BT-TT

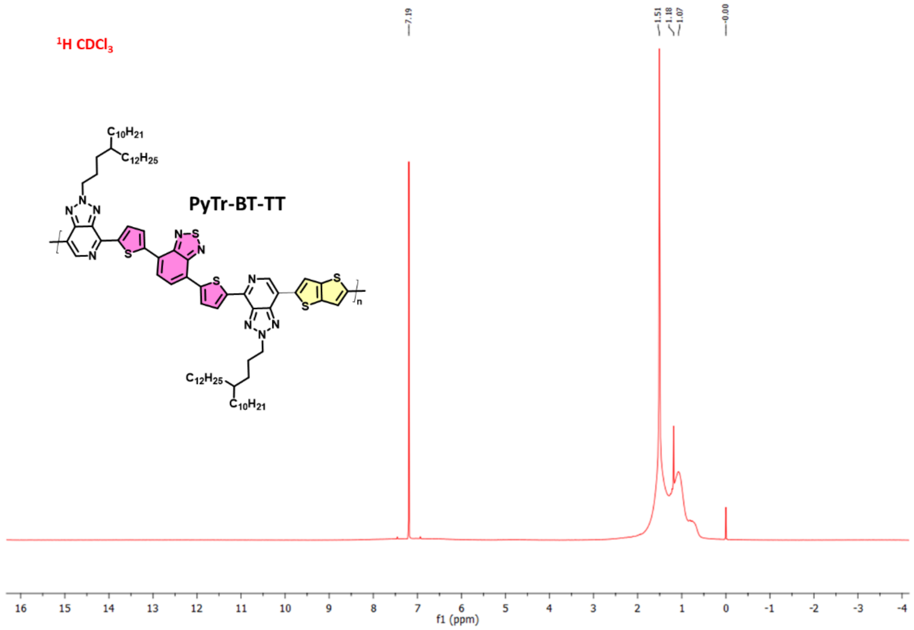

Figure S11: ^{1}H NMR ($CDCl_3$, 298 K, 400 MHz) of PyTr-BT-TT.

3.12 ^{1}H NMR spectra of Polymer PyTr-Th2F2-TT

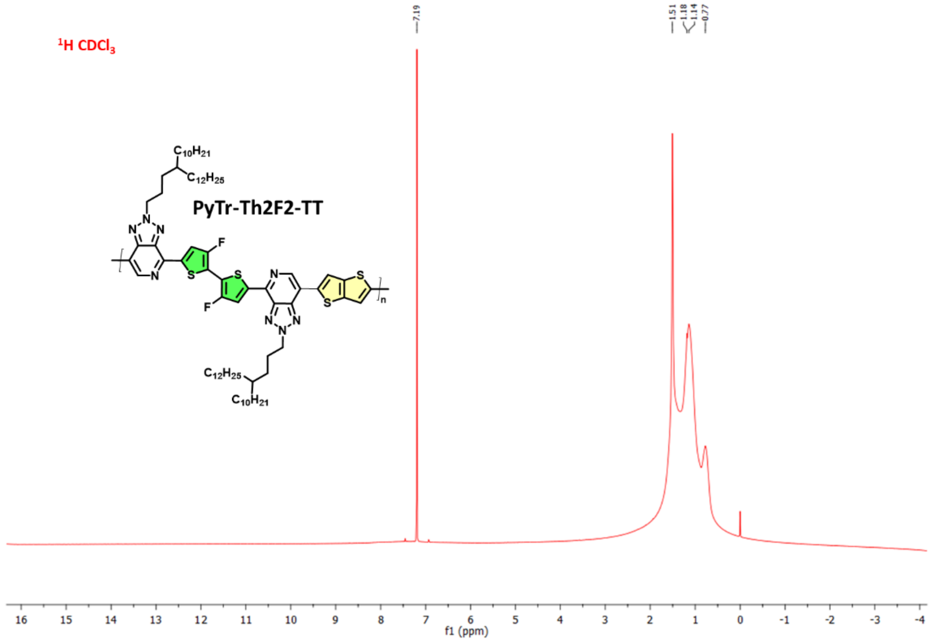

Figure S12: ^{1}H NMR ($CDCl_3$, 298 K, 400 MHz) of PyTr-Th2F2-TT.

4. Gel Permeation Chromatography

4.1 GPC of PyTr-VSe-TT

Single Injection Report Agilent Technologies

**Data file:**	**PyTr-Vse-TT**		
**Sequence Name:**	SingleSample	**Project Name:**	SEC_1260
**Sample name:**	PyTr-VSe-TT_Poly	**Operator:**	SYSTEM
**Instrument:**	SEC_GPC	**Injection date:**	2021-11-01 13:32:55+03:00
**Inj. volume:**	20.000	**Location:**	P2-D1
**Acq. method:**	SKP_new Method31102021.amx	**Type:**	Sample
**Processing method:**	*STD Cali_31102021.pmx	**Sample amount:**	0.00
**Manually modified:**	Manual Integration		

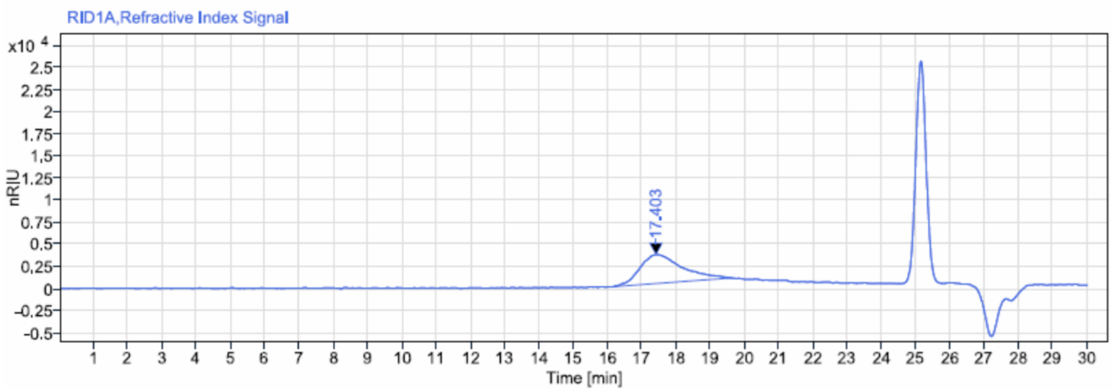

Peak #	Mp (g/mol)	Mn (g/mol)	Mw (g/mol)	Mz (g/mol)	Mz+1 (g/mol)	Mv (g/mol)	PD
1	101097	59493	91551	123798	153967	119322	1.538853

Figure S13: GPC chromatograph of PyTr-VSe-TT.

4.2 GPC of PyTr-BT-TT

Single Injection Report — Agilent Technologies

**Data file:**	**PyTr-BT-TT**		
**Sequence Name:**	SingleSample	**Project Name:**	SEC_1260
**Sample name:**	PyTr-Th-BT-Th-PyTr-TT_Poly_Chloroform	**Operator:**	SYSTEM
**Instrument:**	SEC_GPC	**Injection date:**	2021-11-22 11:39:19+03:00
**Inj. volume:**	20.000	**Location:**	P2-B1
**Acq. method:**	SKP_new Method31102021,amx	**Type:**	Sample
**Processing method:**	STD Cali_31102021_final.pmx	**Sample amount:**	0.00
**Manually modified:**	Manual Integration		

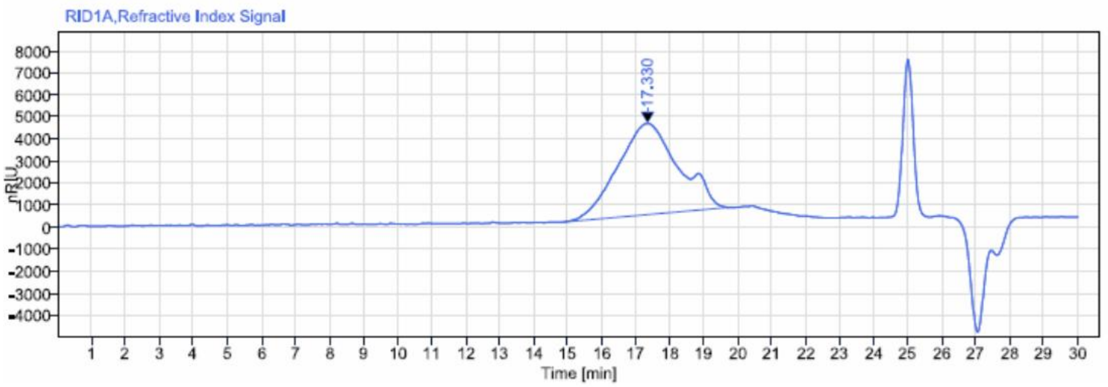

Peak #	Mp (g/mol)	Mn (g/mol)	Mw (g/mol)	Mz (g/mol)	Mz+1 (g/mol)	Mv (g/mol)	PD
1	110840	67014	147656	281075	440857	259608	2,203360

Figure S14: GPC chromatograph of PyTr-BT-TT.

4.3 GPC of PyTr-Th2F2-TT

Single Injection Report — Agilent Technologies

**Data file:**	**PyTr-Th2F2-TT**		
**Sequence Name:**	PyTr-BiThF2-PyTr-TT_Poly_CHCl3	**Project Name:**	SEC_1260
**Sample name:**		**Operator:**	SYSTEM
**Instrument:**	SEC_GPC	**Injection date:**	2021-11-08 12:11:15+03:00
**Inj. volume:**	20.000	**Location:**	P2-B2
**Acq. method:**	SKP_new Method31102021,amx	**Type:**	Sample
**Processing method:**	STD Cali_31102021_final.pmx	**Sample amount:**	0.00
**Manually modified:**	None		

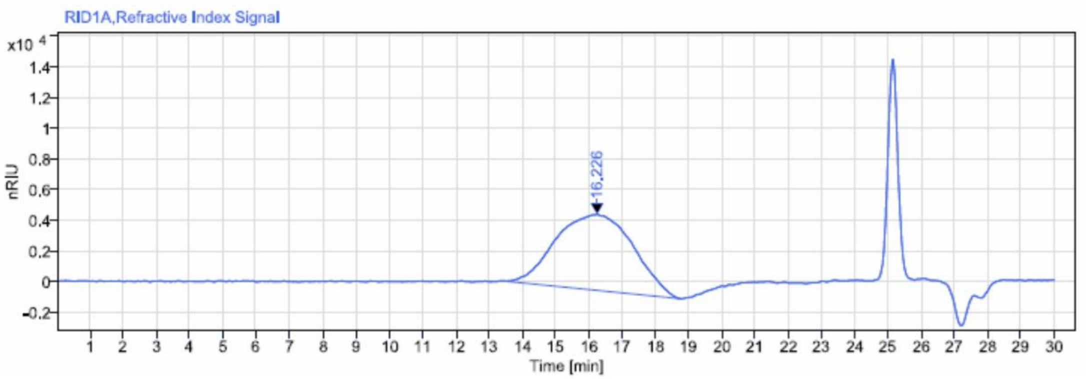

Peak #	Mp (g/mol)	Mn (g/mol)	Mw (g/mol)	Mz (g/mol)	Mz+1 (g/mol)	Mv (g/mol)	PD
1	344748	224351	613642	1375886	2246970	1255956	2,735187

Figure S15: GPC chromatograph of PyTr-Th2F2-TT.

5. Thermal Studies

5.1 TGA analysis

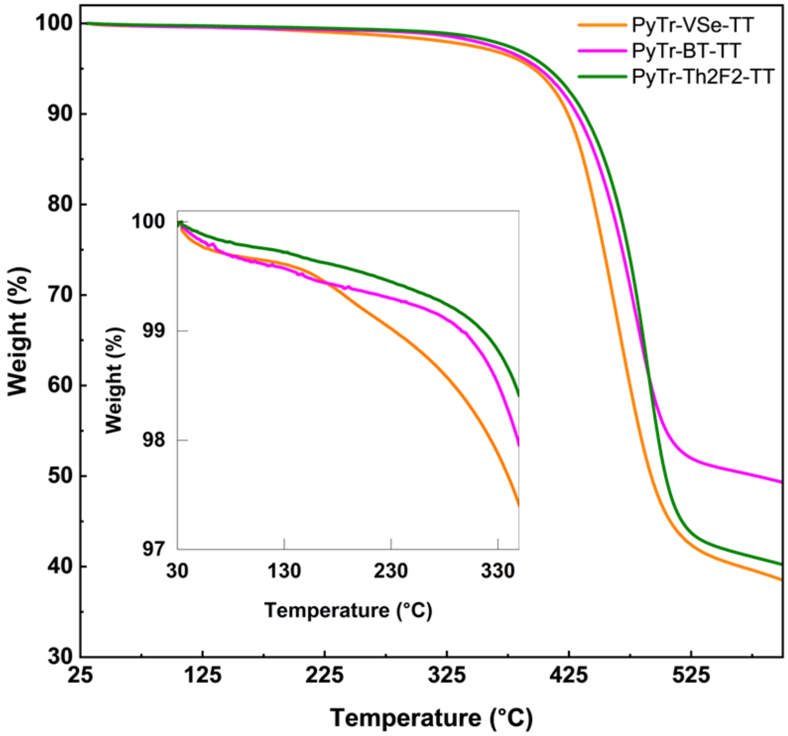

Figure S16: TGA curves of polymers PyTr-VSe-TT, PyTr-BT-TT, and PyTr-Th2F2-TT measured under nitrogen at 10 °C·min^{-1} up to 600 °C using a PerkinElmer instrument. P1: T_{onset} = 414 °C, T_{max} = 464 °C, residue = 38–40%. P2: T_{onset} = 420 °C, T_{max} = 478 °C, residue = 49–50%. P3: T_{onset} = 436 °C, T_{max} = 489 °C, residue = 39–41%.

5.2 DSC analysis

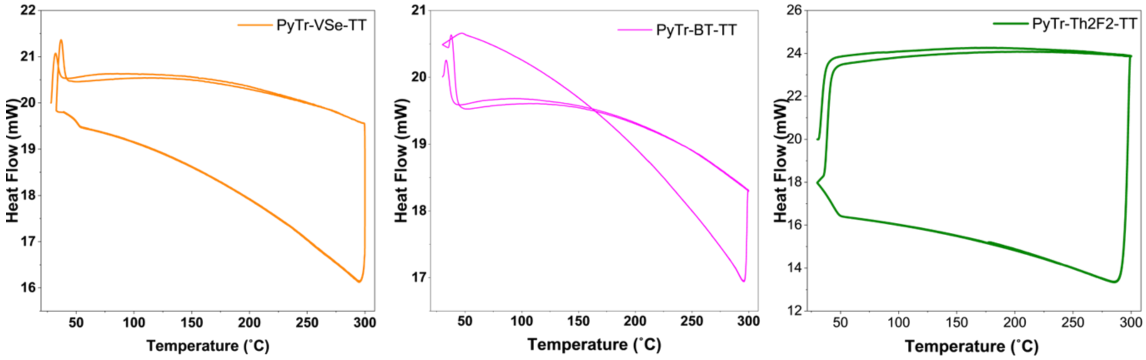

Figure S17: DSC thermogram of PyTr-VSe-TT, PyTr-BT-TT, and PyTrTh2F2-TT showing heat flow (mW) versus temperature (°C) for a sample subjected to two heating and two cooling cycles from 30°C to 300°C at a rate of 10°C/min. Initial endothermic events are visible in the low temperature range, followed by

stable heat flow during subsequent heating and cooling, indicating thermal stability and the absence of major phase transitions.

6. Transistor Characteristics

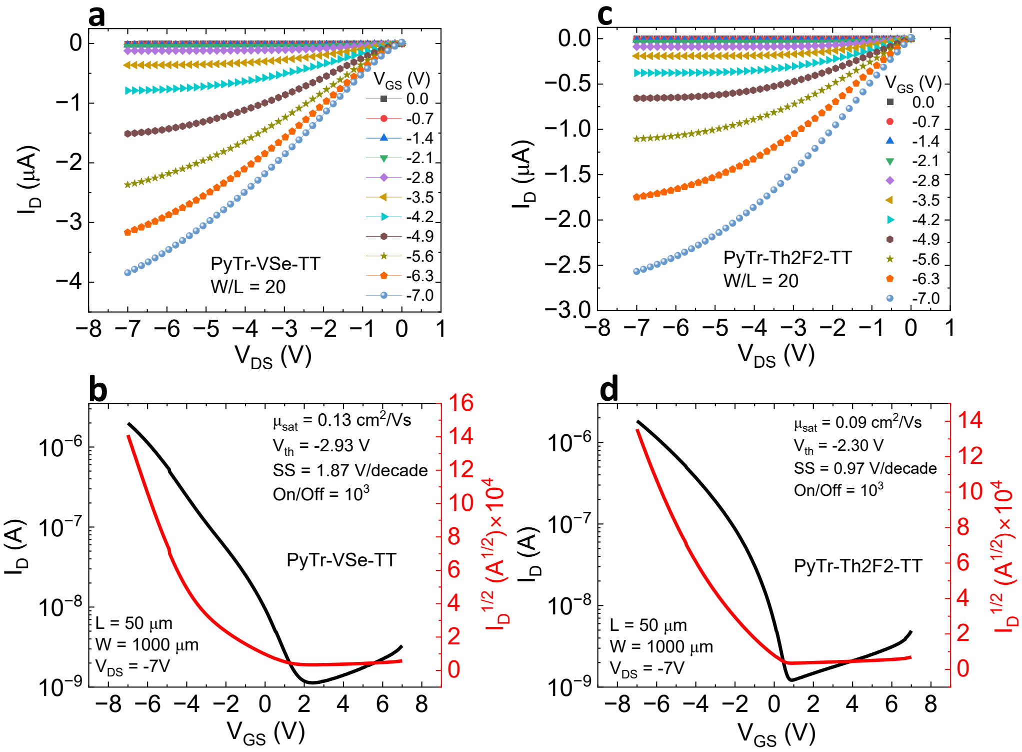

Figure S18: Output and transfer characteristics from PyTr-VSe-TT FET (a, b), PyTr-Th2F2-TT FET (c, d) for W/L ratio of 20.

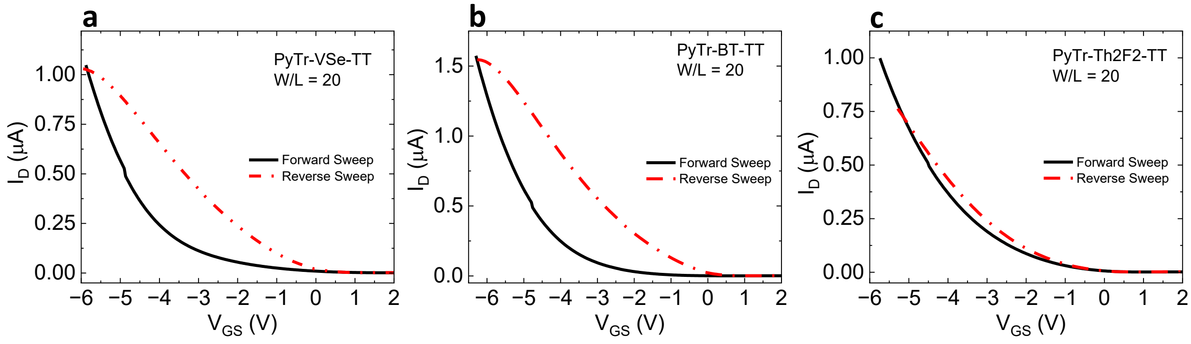

Figure S19: Transfer hysteresis sweeps from representative FETS: (a) PyTr-VSe-TT, (b) PyTr-BT-TT, and (c)PyTr-Th2F2-TT. In each case V_{DS} was set to – 7 V.

7. Synaptic Characteristics

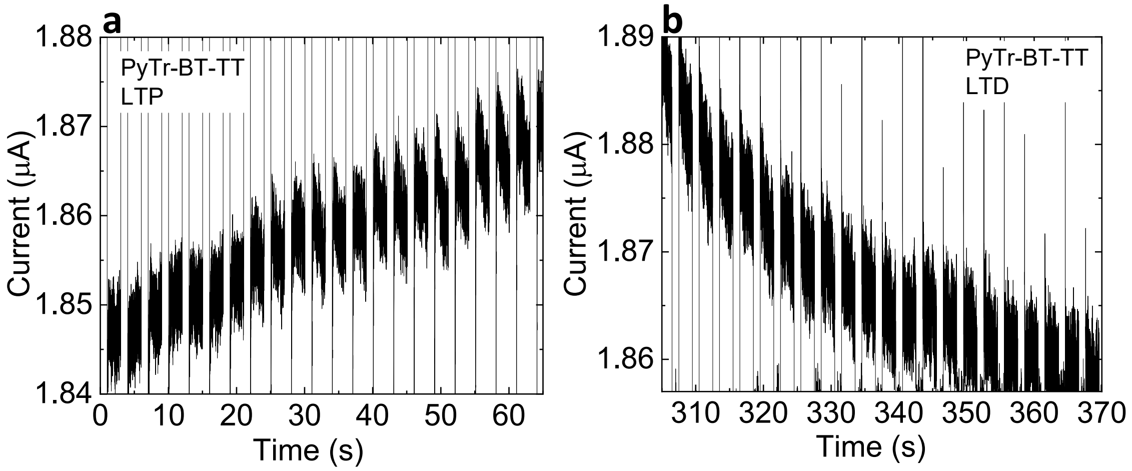

Figure S20: Zoomed-in regions of the postsynaptic output current in a PyTr-BTT-TT/PVDF-HFP FET during LTP (a) and LTD (b).

Table S2: LTP/LTD characteristics of three different PyTr-BT-TT FETs. Run 1 and Run 2 refer to two measurements from the same device over a period of time.

**PyTr-BT-TT FETs**	Device 1 (L = 100 μm)	Device 2; run 1 (L = 50 μm)	Device 2; run 2 (L =50 μm)	Device 3 (L = 75 μm)
β_p (LTP)	3.69	1.89	2.40	4.88
β_d (LTD)	-6.77	-6.77	7.51	-5.72
G_{max}/G_{min}	18.03	17.74	19.01	8.23
Accuracy	74%	76%	65%	72%

8. Trap Density of States Analysis

The trap density of states (DOS) was analyzed using the Grünewald's method.[1-2] The gate-dependent dielectric-semiconductor interface potential $V_0 = V_0(U_g)$ is first determined from:

$$\exp\left(\frac{eV_0}{kT}\right) - \frac{eV_0}{kT} - 1 = \frac{e}{kT}\frac{\varepsilon_i d}{\varepsilon_s l \sigma_0}\left[U_g\sigma(U_g) - \int_0^{U_g} \sigma(\widetilde{U_g})d\widetilde{U_g}\right]. \quad (S1)$$

In Eq. (1) e, k, and T are the elementary charge, the Boltzmann constant, and the absolute temperature, respectively; $U_g = |V_{GS} - V_{FB}|$ and V_{FB} is the flat-band voltage, which is assumed to be the turn-on voltage of the transistor. ε_i and ε_s are the dielectric constant of the ferroelectric

dielectric and the semiconducting layer, respectively. *d* is the thickness of the semiconducting layer and l is the thickness of the dielectric layer. $\sigma(U_g)$, the field effect conductivity is defined as $\sigma(U_g)=L/W\ \ I_D(U_g)/V_{DS}$. σ_0 is the conductivity at U_g = 0. Eq. (S1) is numerically evaluated using the measured field-effect conductivity to determine the interface potential. The solutions of V_0 are used to solve for the carrier density, $p(V_0)$, using a derivative method:

$$p(V_0) = \frac{\varepsilon_0 \varepsilon_i^2}{\varepsilon_s l^2 e} U_{GS} \left(\frac{dV_0}{dU_{GS}}\right)^{-1}, \quad (S2)$$

and the trap DOS (*N(E)*) is then obtain from

$$N(E) \approx \frac{1}{e}\frac{dp(V_0)}{dV_0}. \quad (S3)$$

Eqs (S1-S3) were solved in open-source Python.

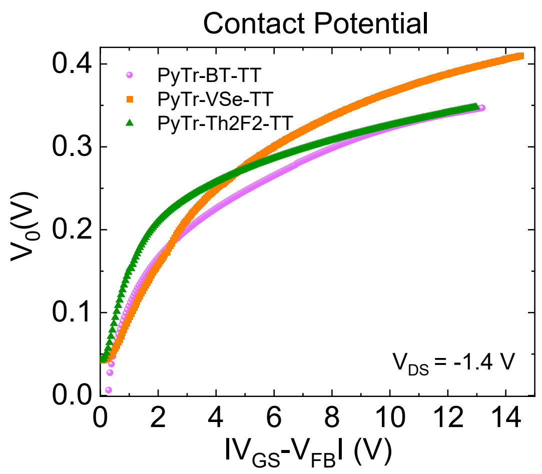

Figure S21: The contact potential vs. $U_g = |V_{GS} - V_{FB}|$ (above flatband) for PyTr-BT-TT, PyTr-VSe-TT, and PyTr-Th2F2-TT/PVDF-HFP FETs.

9. Interface Trap Density (Dit) from Capacitance and Conductance Measurements

The capacitance versus voltage (C-V) and conductance versus voltage (G-V) measurements were carried out from MIS diodes to compare the interface trap density (D_{it}) of the three PyTr copolymers. Most organic semiconductors are typically modeled by the continuum of states model, where the interface traps are assumed to have energy levels that are so closely spaced across the band gap and as such, could be treated as a continuum of states. The equivalent parallel conductance (G_P) using the continuum of states model extracted at different biases from the measured capacitance and conductance is given by:

$$\frac{G_p}{\omega} = \frac{qD_{it}\ln(1+\omega^2\tau^2)}{2\omega\tau}, \qquad (S4)$$

where τ is the interface time constant and $\frac{G_p}{\omega}$ has a maximum at $\omega\tau = 1.98$.

A clear loss peak is seen (in the measured conductance/frequency) suggesting that it is governed by the generation and recombination through interface trap levels. Fig. S2 shows representative C-V and the loss (conductance/angular frequency) versus voltage plots at different frequencies. The D_{it} values were obtained by using Eq. (S4) at different voltages in the transition region.

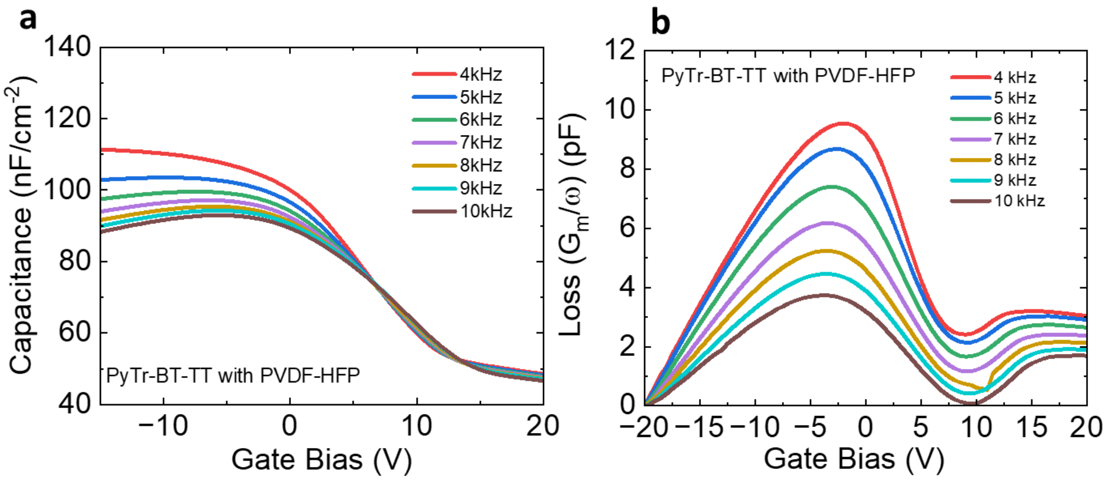

Figure S22: a) C-V and b) G-V curves for PVDF-HFP/PyTr-BT-TT MIS.

10. Normalized Conductance from PyTr-Th2F2-TT/PVDF-TrFE FET

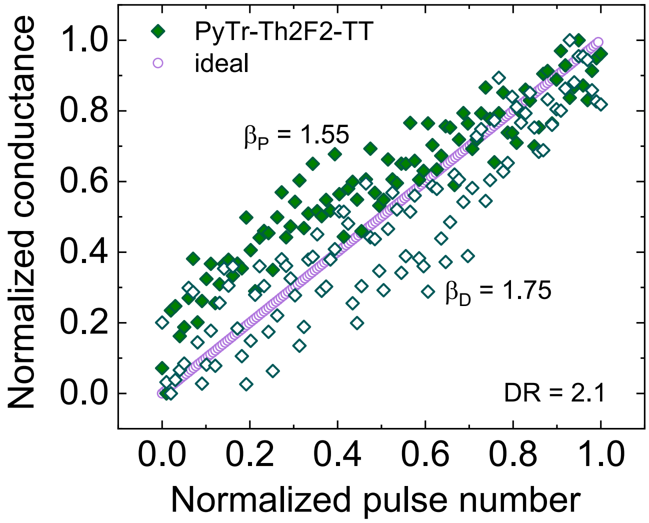

Figure S23: Normalized conductance versus normalized pulse number for a PyTr-Th2F2-TT/PVDF-TrFE FET; the inset shows the synaptic parameters nonlinearity (β_P and B_D) and dynamic range (DR).

11.X-ray Diffraction

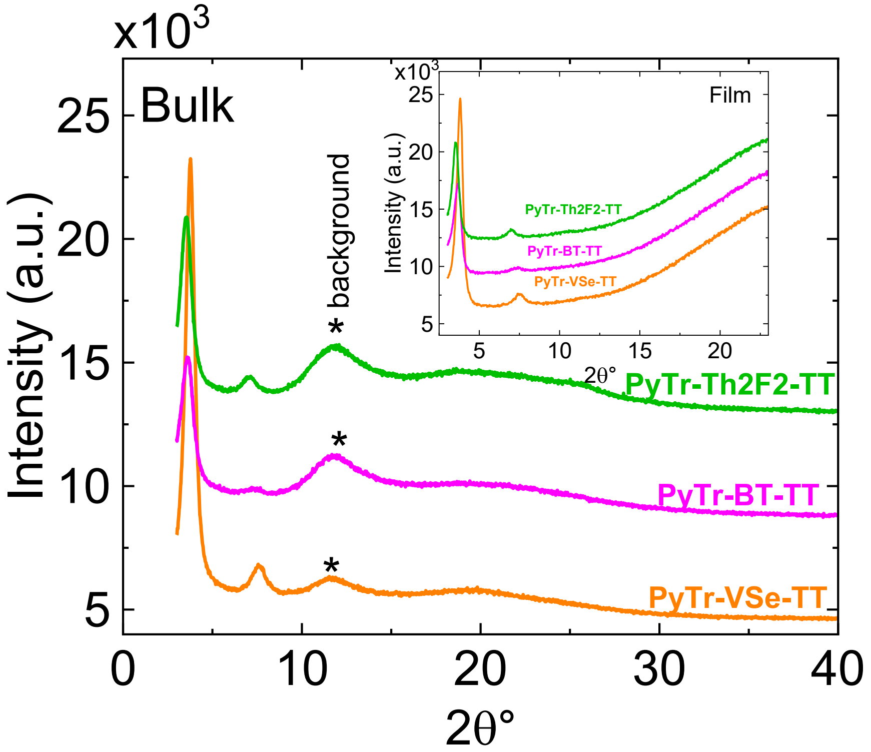

Figure S24: XRD from bulk PyTr-VSe-TT, PyTr-BT-TT, and PyTr-Th2F2-TT. The peak at 11º is from the background glue that was used to affix the samples. The inset shows the XRD data from film samples.

Table S3: The *d* spacing extracted from the XRD data of the three copolymers and the FWHM of the peaks.

	d_1 (Å)	FWHM (°)	d_2 (Å)	FWHM (°)
PyTr-VSe-TT bulk	23.35	0.56	11.68	0.78
PyTr-VSe-TT film	23.09	0.42	11.70	0.69
PyTr-BT-TT bulk	24.13	0.62	12.08	0.79
PyTr-BT-TT film	24.08	0.45	11.93	0.78
PyTr-Th2F2-TT bulk	24.81	0.58	12.36	0.95
PyTr-Th2F2 film	24.79	0.39	12.67	0.64

12. References

1. Grünewald, M.; Thomas, P.; Würtz, D. A Simple Scheme for Evaluating Field Effect Data. *Phys. Status Solidi B* **1980,** *100*, K139-K143.

2. Kalb, W. L.; Batlogg, B. Calculating the Trap Density of States in Organic Field-Effect Transistors from Experiment: A Comparison of Different Methods. *Phys. Rev. B* **2010,** *81*, 035327.
